# Large registry-based analysis of genetic predisposition to tuberculosis identifies genetic risk factors at HLA

**DOI:** 10.1093/hmg/ddac212

**Published:** 2022-08-26

**Authors:** Anniina Tervi, Nella Junna, Martin Broberg, Samuel E Jones, FinnGen, Markku Partinen, Matti Pirinen, Bryan Bryson, Satu Strausz, Hanna-Riikka Kreivi, Caroline A Heckman, Hanna M Ollila

**Affiliations:** Institute for Molecular Medicine Finland, FIMM, HiLIFE, University of Helsinki, Helsinki, Finland; Institute for Molecular Medicine Finland, FIMM, HiLIFE, University of Helsinki, Helsinki, Finland; Institute for Molecular Medicine Finland, FIMM, HiLIFE, University of Helsinki, Helsinki, Finland; Institute for Molecular Medicine Finland, FIMM, HiLIFE, University of Helsinki, Helsinki, Finland; Institute for Molecular Medicine Finland, FIMM, HiLIFE, University of Helsinki, Helsinki, Finland; Department of Pulmonology, University of Helsinki and Helsinki University Hospital, Helsinki, Finland; Institute for Molecular Medicine Finland, FIMM, HiLIFE, University of Helsinki, Helsinki, Finland; Institute for Molecular Medicine Finland, FIMM, HiLIFE, University of Helsinki, Helsinki, Finland; Department of Anesthesia, Critical Care and Pain Medicine, Massachusetts General Hospital and Harvard Medical School, Boston, MA, USA; Program in Medical and Population Genetics, Broad Institute, Cambridge, MA, USA

## Abstract

Tuberculosis is a significant public health concern resulting in the death of over 1 million individuals each year worldwide. While treatment options and vaccines exist, a substantial number of infections still remain untreated or are caused by treatment resistant strains. Therefore, it is important to identify mechanisms that contribute to risk and prognosis of tuberculosis as this may provide tools to understand disease mechanisms and provide novel treatment options for those with severe infection. Our goal was to identify genetic risk factors that contribute to the risk of tuberculosis and to understand biological mechanisms and causality behind the risk of tuberculosis. A total of 1895 individuals in the FinnGen study had International Classification of Diseases-based tuberculosis diagnosis. Genome-wide association study analysis identified genetic variants with statistically significant association with tuberculosis at the human leukocyte antigen (HLA) region (*P* < 5e−8). Fine mapping of the HLA association provided evidence for one protective haplotype tagged by HLA DQB1^*^05:01 (*P* = 1.82E−06, OR = 0.81 [CI 95% 0.74–0.88]), and predisposing alleles tagged by HLA DRB1^*^13:02 (*P* = 0.00011, OR = 1.35 [CI 95% 1.16–1.57]). Furthermore, genetic correlation analysis showed association with earlier reported risk factors including smoking (*P* < 0.05). Mendelian randomization supported smoking as a risk factor for tuberculosis (inverse-variance weighted *P* < 0.05, OR = 1.83 [CI 95% 1.15–2.93]) with no significant evidence of pleiotropy. Our findings indicate that specific HLA alleles associate with the risk of tuberculosis. In addition, lifestyle risk factors such as smoking contribute to the risk of developing tuberculosis.

## Introduction

Tuberculosis (TB) is an infectious disease caused by *Mycobacterium tuberculosis*. The bacteria are transmitted via airborne transmission mainly affecting the pulmonary organs (pulmonary TB), but they can also affect other organs (extrapulmonary TB) (1,2). TB can manifest as a latent non-transmissible form or as an active transmissible form where patients experience symptoms such as high fever, persistent cough, fatigue and weight loss (1). Most individuals infected by *M. tuberculosis* will not develop TB, whereas 5–10% of infected individuals develop the disease. Individuals with a compromised immune system, such as those infected with HIV (human immunodeficiency virus), have a higher likelihood of developing TB (2).

Incidence rates of TB in most of Europe and Northern America are less than 10 per 100 000 per year, and mortality in HIV-negative people is less than 5 per 100 000 per year (2). After development of BCG (Bacillus Calmette–Guérin) vaccination in 1921, people were widely vaccinated in Europe, which lead to declining TB incidence rates. Decline was further assisted by development of antibiotic treatments in the 1940s and 1950s, after which latent TB infections could be treated (3,4). However, Europe has the highest rate of new reported cases of multidrug-resistant TB (2).

The risk for TB is also highly correlated with comorbid diseases. While HIV infection is the strongest risk factor for TB disease, TB infection also exacerbates the course of HIV infection and is associated with 4-fold all-cause mortality among HIV patients (5). Similarly, malnutrition as well as deficiency of vitamins C or D has been associated with TB disease although this association is also related with overall fragility: very young and very old people have elevated TB risk (6–8). Finally, smoking increases risk for latent TB infection and TB disease more than 2-fold (9), and public health campaigns against smoking have been applied in high-risk areas for TB.

On a global scale, WHO estimated that 10 million people developed TB disease in the year 2019 and from those estimated 1.2 million HIV-negative individuals died due to TB in 2019 (2). Although there are treatments for TB, long-term consequences such as pulmonary dysfunction and prolonged respiratory symptoms may expose survivors to other respiratory disorders, for instance, to chronic obstructive pulmonary disease (COPD) (10–13). In addition, treatment-resistant strains and comorbidity burden create challenges in managing TB at the local and global scale.

Therefore, it is crucial to understand the biological mechanisms that contribute to the onset of TB. Understanding these mechanisms is likely to result in better disease management and potentially novel treatment options as well as non-pharmaceutical measures to prevent and to treat TB infections.

By using genetic tools, we are able to study the biological mechanisms underlying TB along with causal relationships between TB and different comorbidities. Host genetic factors in TB have been studied in various different populations (14–24). In European ancestry *M. tuberculosis* or pulmonary TB genome-wide association study (GWAS), three variants rs557011, rs9271378 and rs9272785 in the human leukocyte antigen (HLA)-region reached genome-wide significance (*P* < 5E−08) (18). Additionally, in self-reported positive TB test result GWAS, variant rs2894257 (also from the HLA region) was found to be genome-wide significant in European ancestry individuals (19).

In this study, we used data from the FinnGen study data release 7 (R7) to explore the biological mechanisms underlying TB in 310 000 individuals. We were especially interested in assessing host genetic components using GWAS, and exploring comorbidity burden using genetic correlations, epidemiological tools, and causality with Mendelian randomization (MR).

## Results

### Genome-wide association study

**Figure 1:**
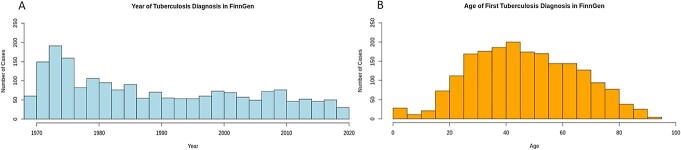
FinnGen R7 distribution of tuberculosis diagnosis and age at first diagnosis. (**A**) A bar chart illustrating diagnosed tuberculosis cases by year of diagnosis in FinnGen R7 starting from 1970 (start of the Care Register for Health Care Inpatient Visits in Finland) until the end of 2019 (end of follow-up time December 31, 2019). (**B**) A bar chart illustrating diagnosis age in individuals with tuberculosis (age range is from birth to 100 years of age).

**Figure 2:**
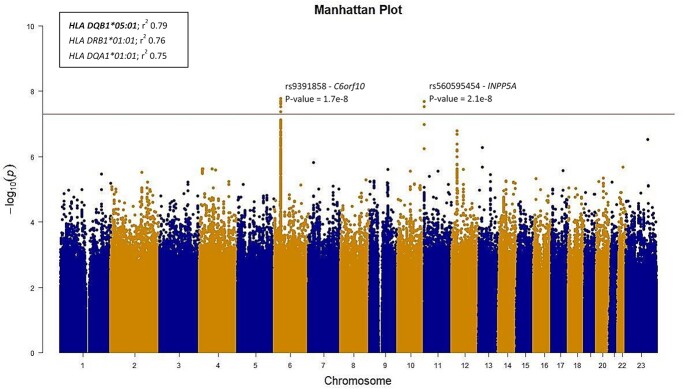
A Manhattan plot from a tuberculosis GWAS in FinnGen R7. (**A**) Manhattan plot from GWAS in 1895 individuals with ICD-based tuberculosis and 307 259 controls from FinnGen R7 where each dot represents an SNP from chromosome 1–23. The lead variants, rs9391858 and rs560595454, and their respective *P*-values are marked to their corresponding positions. *r*^2^ values represent the linkage disequilibrium (LD) between the lead variant (rs9391858) and the respective HLA alleles.

To study the host genetic components contributing to TB, we performed GWAS in 1895 individuals with ICD-based TB and 307 259 controls from FinnGen R7 (Table 1, Fig. 1). We identified four genome-wide significant (*P* < 5E−08) single nucleotide polymorphisms (SNPs) of which two are at the HLA locus in the chromosomal position 6p21 (rs9391858 and rs33915496, *r*^2^ = 0.14) and two are located in chromosome 10 (rs560595454 and rs562763274) in gene *INPP5A* (inositol polyphosphate-5-phosphatase A) (Fig. 2). The lead SNP (rs9391858) was closest to and downstream from gene *C6orf10* (open reading frame on chromosome 6*,* also known as *TSBP1* (testis expressed basic protein 1)), which is located in the HLA locus and upstream from *HLA DRA* gene (Supplementary Material, Figs S1 and S2). We also performed GWAS on subsets of TB endpoints in FinnGen, respiratory TB (1380 cases and 307 312 controls) and TB of other organs (538 cases and 307 259 controls), where a genome-wide significant SNP was identified in respiratory TB GWAS in the HLA locus in between *HLA DRA* and *HLA DQA1* genes and with high linkage disequilibrium (LD) with rs33915496 (rs2395516, *P* = 4.5E-09, *r*^2^ = 0.90) (Supplementary Material, Figs S3 and S4).

**Table 1:** FinnGen R7 metrics for tuberculosis

	Tuberculosis	Controls	FinnGen R7
N-number	1895	307 259	309 154
Age of onset (mean)	46.56 (SD = 18.71)		
Sex (male)	1118 (59.0%)	134 290 (43.7%)	135 408 (43.8%)
BMI (mean)	26.47 (SD = 4.93)	27.36 (SD = 5.42)	27.35 (SD = 5.41)

The number of tuberculosis (all diagnosis subcodes) in FinnGen cohort R7, number of controls and the total number of individuals in FinnGen R7 are reported. The mean age of first onset of tuberculosis, number of males in cases, controls and in FinnGen R7, and mean BMI are also reported.

To understand if the risk variants reflect a shared effect across populations, we examined the variants in the Biobank Japan (BBJ) and in the UK Biobank (UKB). The lead variants replicated in the BBJ for respiratory (pulmonary) TB (rs9391858: *P* = 7.5e−4, beta [SE] = −0.10 [0.03] and rs33915496: *P* = 3.2e−13, beta [SE] = 0.12 [0.02]) (24). Furthermore, the effects were in the same direction in the UKB, but did not reach statistical significance (rs9391858: *P* = 0.16, beta [SE] = −0.05 [0.03], and rs33915496: *P* = 0.35, beta [SE] = 0.02 [0.03]). Finally, meta-analysis across replication cohorts for pulmonary TB supported our HLA association although there was notable between-study heterogeneity (rs140780894: *P* = 3.2e-23, beta [SE] = 0.19 [0.02]) (Supplementary Material, Figs S5 and S6, Supplementary Material, Tables S1 and S2).

In previous studies other genes, such as *ASAP1* (ArfGAP with SH3 domain, ankyrin repeat and PH domain 1), *IL9* (interleukin 9), *WT1* (WT1 transcription factor), *ADAM12* (ADAM metallopeptidase domain 12) and *MFN2* (mitofusin 2)*,* have been shown to associate with TB (16,17,20–22). In the FinnGen R7 cohort, we were able to replicate all the earlier reported risk variants with *P* < 0.01 level (rs77668120 (*P* = 6.3E−05), rs1403525097 (*P* = 4.4E−03), rs180917274 (*P* = 6.7E−03), rs145176301 (*P* = 7.7E−04) and rs7530770 (*P* = 8.1E−03), respectively). Similarly, we replicated all of the previously reported variants that have been reported in European ancestry TB GWAS (rs557011 (*P* = 3.1E−05), rs9271378 (*P* = 8.5E−06) and rs2894257 (*P* = 2.6E−02)). Additionally, homozygosity for *TYK2* P1104A variant has been reported as a risk factor for TB previously in the UKB (25). In our current study, the homozygosity of *TYK2* P1104A variant was close to significance although we were not able to replicate it in the current study (*P* = 0.091).

### HLA fine mapping

**Figure 3:**
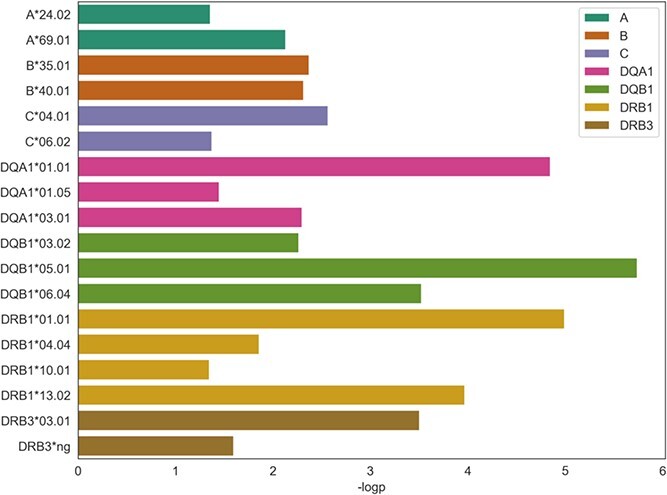
HLA fine mapping. Association of HLA alleles with tuberculosis in FinnGen R7 (cut off *P*-value 0.05).

Traditionally, alleles at HLA class II are related to transplantation outcomes, autoimmune and infectious diseases. Therefore, we used imputed HLA allele information to assess if alleles in addition to individual variants associate with TB using multivariate logistic regression adjusting for age at death or at end of follow-up (December 31, 2019), sex and the first 20 genetic principal components. We found three associations of protective effect estimate with HLA alleles: HLA DQB1^*^05:01 (*P* = 1.82E-06, OR = 0.81 [CI 95% 0.74–0.88]), HLA DRB1^*^01:01 (*P* = 6.86E−06, OR = 0.82 [CI 95% 0.75–0.89]) and HLA DQA1^*^01:01 (*P* = 1.004E−05, OR = 0.82 [CI 95% 0.75–0.90]) (Fig. 3, Supplementary Material, Tables S3 and S4). Due to the high allelic diversity and high LD at the HLA region (26), we measured the LD between lead variant rs9391858 at the HLA locus and individual HLA-alleles using r^2^ measure (27). HLA-alleles HLA DQB1^*^05:01, HLA DRB1^*^01:01 and HLA DQA1^*^01:01 were in high LD with the lead SNP rs9391858 (*r*^2^ = 0.79, 0.76 and 0.75, respectively) (Fig. 2). To test if the signal was independent, we computed conditional association statistics adjusting for the lead variant rs9391858. HLA DQB1^*^05:01, HLA DRB1^*^01:01 and HLA DQA1^*^01:01 were not significant after conditioning for the lead SNP suggesting that the lead SNP and the HLA alleles reflect the same signal (Supplementary Material, Table S5). Furthermore, we repeated the multivariate logistic regression analysis with the respiratory TB phenotype and the lead alleles remained the same (Supplementary Material, Table S6).

In addition, we identified three HLA alleles that show evidence of increasing TB risk: HLA DRB1^*^13:02 (*P* = 1.07E−04, OR = 1.35 [CI 95% 1.16–1.57]), HLA DQB1^*^06:04 (*P* = 2.98E−04, OR = 1.34 [CI 95% 1.14–1.56]) and HLA DRB3^*^03:01 (*P* = 3.13E−04, OR = 1.33 [CI 95% 1.14–1.56]) (Supplementary Material, Table S2). Unlike HLA DQB1^*^05:01, the risk alleles were not in high LD with risk SNP rs9391858 (*r*^2^ = 0.2) or with HLA DQB1^*^05:01. After stepwise logistic regression adjusting the HLA associations with the lead allele HLA DQB1^*^05:01, these three positive effect alleles remained statistically significant (*P* = 0.0007, 0.002, 0.002, respectively) (Supplementary Material, Table S11).

### Epidemiological and genetic correlates

**Figure 4:**
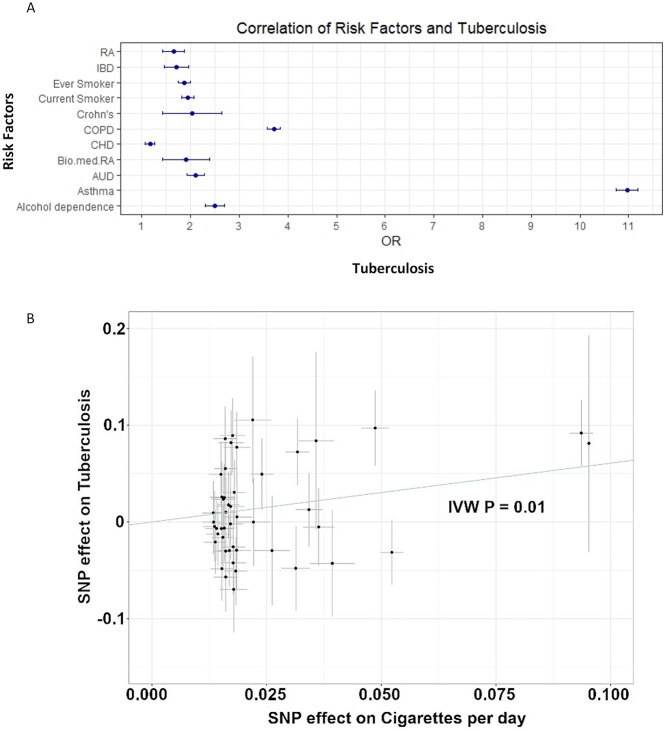
Error bar plot on association of known risk factors with tuberculosis and Mendelian randomization analysis (MR) with smoking trait. (**A**) Bar plot demonstrates odds ratios (OR) and their respective 95% confidence intervals between tuberculosis and risk factors. From the studied risk factors (Supplementary Material, Table S5), RA (rheumatoid arthritis), IBD (inflammatory bowel disease), ever smoker, current smoker, Crohn’s disease, COPD (chronic obstructive pulmonary disease), CHD (major coronary heart disease event), biological medication for rheumatoid arthritis (Bio.med. RA), AUD (alcohol use disorder), asthma and alcohol dependence had statistically significant and positive association with tuberculosis. (**B**) MR suggests habitual smoking (instrumented by cigarettes per day) as a risk factor for tuberculosis (inverse-variance weighted *P* = 0.01). The increase of habitual smoking increases the risk for tuberculosis.

To study the association between known risk factors and TB, we used multivariate logistic regression and adjusted for age at death or end of follow-up (December 31, 2019), sex, body mass index (BMI) and the first 10 genetic principal components. Positive correlation with TB was witnessed in current smoking status (*P* = 2.0E−16, OR = 1.94 [CI 95% 1.73–2.18]), ever smokers (*P* = 2.0E−16, OR = 1.87 [CI 95% 1.66–2.12]), rheumatoid arthritis (RA) (*P* = 1.1E−05, OR = 1.65 [CI 95% 1.32–2.07]), inflammatory bowel disease (IBD) (*P* = 4.5E−05, OR = 1.71 [CI 95% 1.32–2.21]), Crohn’s disease (*P* = 2.0E−02, OR = 2.04 [CI 95% 1.43–2.65]), COPD (*P* = 2.0E−16, OR = 3.71 [CI 95% 3.26–4.22]), major coronary heart disease event (CHD) (*P* = 3.8E−03, OR = 1.17 [CI 95% 1.05–1.30]), biological medication for RA (*P* = 1.1E−02, OR = 1.91 [CI 95% 1.42–2.40]), asthma (*P* = 2.0E−16, OR = 10.97 [CI 95% 10.75–11.19]), alcohol use disorder (AUD) (*P* = 1.1E−15, OR = 2.11 [CI 95% 1.76–2.53]) and alcohol dependence (*P* = 2.0E−16, OR = 2.50 [CI 95% 2.04–3.06]) (Fig. 4A and Supplementary Material, Table S7).

To explore the effect on survival, we used the Kaplan–Meier estimator and Cox proportional hazards model to see the effect of TB and its known comorbidities on survival. Kaplan–Meier estimator indicated shorter lifespan within TB cases that either are current smokers or have COPD, alcohol dependence or AUD diagnosis (Supplementary Material, Figs S7 and S8, Supplementary Material, Figs S10 andS11). Furthermore, Cox proportional hazards model showed associations between survival and current smoking status (*P* = 3.27E−07, hazard ratio (HR) = 2.46 [CI 95% 1.74–3.48]), COPD (*P* = 8.98E−10, HR = 1.73 [CI 95% 1.45–2.06]), alcohol dependence (*P* = 4.20E−07, HR = 2.24 [CI 95% 1.64–3.06]) and AUD (*P* = 1.45E-06, HR = 2.03 [CI 95% 1.52–2.71]) within TB patients (Supplementary Material, Table S8). Other tested comorbidities did not have an effect on survival within TB patients (Supplementary Material, Table S8). In addition, Kaplan–Meier estimator indicated shorter median survival time in non-smoking TB patients compared to smoking patients (Supplementary Material, Fig. S9). We recognize that the FinnGen study may be enriched for clinical cases and effects with comorbidities may not reflect the association at population level. We therefore computed subset analysis in population sample subset of the FinnGen study (FINRISK samples: https://thl.fi/en/web/thlfi-en/research-and-development/research-and-projects/the-national-finrisk-study) that reflect better the population structure of Finland. We observed with Cox proportional hazards model that in this population smoking (ever smoker) affected the survival (*P* = 2.00E−16, HR = 1.76 [CI 95% 1.64–1.89]) as did COPD (*P* = 2.00E−16, HR = 2.04 [CI 95% 1.84–2.26]), alcohol dependence (*P* = 2.00E−16, HR = 3.22 [CI 95% 2.83–3.65]) and AUD (*P* = 2.00E−16, HR = 3.05 [CI 95% 2.74–3.41]), but TB did not (*P* = 0.12). Furthermore, we did not see significant interaction between smoking and TB, COPD and TB, alcohol dependence and TB or AUD and TB on survival (*P*-value interaction 0.85, 0.42, 0.47 and 0.59, respectively).

In addition to association studies, we estimated the genetic overlap between TB and the risk factors identified from epidemiological analysis using LD score regression. In agreement with the epidemiological associations, smoking measured as number of cigarettes previously smoked daily (*r_g_* = 0.4377, *P* = 0.003) and current tobacco smoking (*r_g_* = 0.3476, *P* = 0.0048) were positively associated with TB (Supplementary Material, Tables S10 and S12).

To explore causality between TB and different traits, we performed MR analysis. MR suggested smoking as a risk factor for TB (inverse-variance weighted *P* = 0.01, OR = 1.83 [CI 95% 1.15–2.93]) with no significant pleiotropy found (Egger intercept pleiotropy test *P* = 0.46) (Fig. 4B). In addition, we tested known risk factors, BMI, vitamin D deficiency, COPD and AUD with TB but found no causality between these traits in the FinnGen R7 cohort.

## Discussion

In this paper, we identified genetic variants from the HLA region that protect from TB. In addition, we identified associations with comorbidities, where smoking and alcohol dependence, in particular, associated with TB. Our results indicate a unique interplay between host genetic components, primarily from the HLA DQB1^*^05:01 as a protective factor, and HLA DRB1^*^13:02 as susceptibility factor to TB. In addition, our findings highlight the environmental contribution from lifestyle and comorbid factors including smoking and alcohol dependence with TB susceptibility and survival.

Our findings align with previous studies showing the association between HLA class II region and TB (see supplemental information for more detailed description) (14,15,18,19,21,23,24,28–35). Other genes shown to be associated with TB susceptibility in previous studies, such as *ASAP1* (17), did not reach genome-wide significance in our study. In addition to HLA class II associations in TB, a recent integrative genomic analysis combining different data sets identified overall 26 candidate genes associated with TB susceptibility (36). This earlier evidence and our findings indicate that host and pathogen genetic factors affect disease susceptibility and severity.

There is clear heterogeneity between HLA class II allele association in different populations and different lead variants associate in different countries or ethnic groups. The reason behind the heterogeneity is unknown but may be due to selection, a bottleneck effect, pathogen-driven diversity or altering virulence of different lineages of *M. tuberculosis* bacteria (37–41). Furthermore, HLA alleles show diversity and individual allele frequencies differ across populations, which affects the power to observe association in different populations for those alleles that are less common. In functional studies, the role of HLA class II genes in TB has started to spark interest. Kust *et al*. showed increased level of natural killer cells expressing *HLA DR* gene in the blood of primary diagnosed TB (42). Furthermore, Tippalagama *et al*. found that the number of *HLA DR* positive circulating CD4 T-cells was increased in patients with active form of TB (43). The *HLA DRB1* gene specifically has been suggested to modulate cytokine responses to *M. tuberculosis* and thus modulate the immune response of an individual to the infection (44).

In addition to HLA class II association with TB, we report the contribution of smoking and alcohol dependence to TB. Smoking is already a well-established risk factor in TB (45). Our results not only show a significant epidemiological association with TB, smoking habits and smoking-related disease (COPD), but also show genetic correlation and causality between these traits. Through MR we identified causal relationship where increase in habitual smoking increases the risk for TB. In the FinnGen cohort, TB patients were enriched for smoking throughout different decades starting from the 1970s (Supplementary Material, Table S9). All of these gained results indicate that smoking is a major risk factor for TB, alongside severe alcohol usage.

Our study does have some limitations. The lead SNP (rs9391858) identified by our GWAS was located nearest to the gene *HLA DRA* among the HLA genes. However, in the HLA fine mapping analysis *HLA DRA* was not among the imputed HLA genes in FinnGen. We used LD score regression to estimate the genetic correlation between different traits and TB. Our LD score regression showed low heritability within traits (1–5%), which affects the reliability of those results and therefore they should be interpreted with caution. Low heritability was most likely due to the fact that the HLA region was removed from the analysis and our results were mainly from that specific chromosomal region. Our survival analysis for epidemiological traits and TB was conducted using a Cox proportional hazards model that assumes all used traits being constant over time. We validated our Cox model, evaluating the proportionality of the predictors against time. The results showed slight statistical significance (*P* = 0.04), which indicates that not all predictors met the proportional assumption of the Cox model, with smoking status being one of the most evident one from the included predictors (smoking *P* = 0.039) (Supplementary Material, Table S8, global *P*-value). Furthermore, Cox proportional hazards model only within TB patients can introduce collider bias and therefore the results should be interpreted with that kept in mind. Nevertheless, smoking was associated with TB in our causality estimates and risk factor correlations that highlight smoking being a significant risk factor in TB. Our endpoints in FinnGen for TB were defined using ICD-code based diagnoses (TB: ICD-10 codes A15–A19; respiratory TB: A15–A16; TB of other organs: A17–A19). Unfortunately, we did not have information on individuals among controls who would have been infected by *M. tuberculosis* and might suffer from an undiagnosed latent TB infection. Additionally, we assumed in our analysis BMI and smoking information to remain unchangeable throughout the studied period due to the longitudinal nature of the data used, which does not necessarily represent the BMI and smoking information at the time of the TB diagnoses.

In Finland, cases of TB have gradually decreased from the mid-20th century to present day (46). Almost half of the TB cases in Finland (year 2018) are witnessed among immigrants and transmission of TB is rare between immigrants and Finnish-born individuals (46–48). Most of the Finnish-born cases encountered present day are elderly individuals with reactivation of a latent TB originally acquired during their childhood (46). In our study, FinnGen participants were matched against a Finnish reference panel, which highlights our genetic findings to be specific to individuals of Finnish ancestry and can be also regarded as limiting factor in our study. Furthermore, it has been previously shown that individuals with RA in Finland have a higher incidence of TB compared to the general Finnish population (49). This was also witnessed in the FinnGen cohort alongside other comorbidities and risk factors that are known risk factors for TB in other populations as well (8).

Our results highlight the importance of host genetic factors in TB alongside environmental risk factors. These additional results may benefit research in and, ultimately, clinical interventions for TB and other infectious diseases. However, further epidemiological and functional studies are needed to reveal the biological mechanisms underlying the individual reaction we have as humans to different infections.

## Materials and Methods

### Study cohorts

The FinnGen study (https://www.finngen.fi/en) is a public–private partnership including Finnish universities, biobanks and hospital districts together with several pharmaceutical companies founded in the year 2017. The aim is to collect both National Health Record and genetic data from 500 000 Finns. The study participants include patients with acute and chronic diseases as well as healthy voluntary and population collections. R7 includes ∼310 000 individuals (~175 000 females and ~135 000 males).

The UKB is a prospective study containing over 500 000 individuals of mainly European ancestry (50). Invited participants were aged between 37 and 73 years upon entry to the study between 2006 and 2010 and were residents of the UK. The UKB combines medical health record data, lifestyle measures, questionnaire data, genotypes, blood count data and biochemistry measures, among other data. The electronic health records of UKB are a combination of Hospital Episode Statistics in-patient (HES; max. *N* = 440 512) and primary care (GP; max. *N* = 231 364) data. These data are updated frequently in order to capture the health trajectories of the participants.

The BBJ project is a prospective cohort based on Japanese hospital records, which was launched in 2003 (51). Cohort data consist of DNA samples, serum samples and clinical data from 200 000 participants gathered from 66 hospitals nationwide. Registration for the cohort happened between the years of 2003 and 2008 and the data were updated annually until 2013 with interviews and medical records reviews.

### FinnGen ethics statement

Patients and control subjects in FinnGen provided informed consent for biobank research, based on the Finnish Biobank Act. Alternatively, separate research cohorts, collected prior the Finnish Biobank Act came into effect (in September 2013) and start of FinnGen (August 2017), were collected based on study-specific consents and later transferred to the Finnish biobanks after approval by Fimea (Finnish Medicines Agency), the National Supervisory Authority for Welfare and Health. Recruitment protocols followed the biobank protocols approved by Fimea. The Coordinating Ethics Committee of the Hospital District of Helsinki and Uusimaa (HUS) statement number for the FinnGen study is Nr HUS/990/2017.

The FinnGen study is approved by Finnish Institute for Health and Welfare (permit numbers: THL/2031/6.02.00/2017, THL/1101/5.05.00/2017, THL/341/6.02.00/2018, THL/2222/6.02.00/2018,THL/283/6.02.00/2019, THL/1721/5.05.00/2019 and THL/1524/5.05.00/2020), Digital and population data service agency (permit numbers: VRK43431/2017–3, VRK/6909/2018–3, VRK/4415/2019–3), the Social Insurance Institution (permit numbers: KELA 58/522/2017, KELA 131/522/2018,KELA 70/522/2019, KELA 98/522/2019, KELA 134/522/2019, KELA 138/522/2019, KELA 2/522/2020, KELA 16/522/2020), Findata permit numbers THL/2364/14.02/2020, THL/4055/14.06.00/2020, THL/3433/14.06.00/2020, THL/4432/14.06/2020, THL/5189/14.06/2020, THL/5894/14.06.00/2020, THL/6619/14.06.00/2020, THL/209/14.06.00/2021, THL/688/14.06.00/2021, THL/1284/14.06.00/2021,THL/1965/14.06.00/2021, THL/5546/14.02.00/2020 and Statistics Finland (permit numbers: TK-53-1041-17 and TK/143/07.03.00/2020 (earlier TK-53-90-20)).

The Biobank Access Decisions for FinnGen samples and data utilized in FinnGen Data Freeze7 include: THL Biobank BB2017_55, BB2017_111, BB2018_19, BB_2018_34, BB_2018_67, BB2018_71, BB2019_7, BB2019_8, BB2019_26, BB2020_1, Finnish Red Cross Blood Service Biobank 7.12.2017, Helsinki Biobank HUS/359/2017, Auria Biobank AB17–5154 and amendment #1 (August 17 2020), Biobank Borealis of Northern Finland_2017_1013, Biobank of Eastern Finland 1186/2018 and amendment 22 §/2020, Finnish Clinical Biobank Tampere MH0004 and amendments (21.02.2020 06.10.2020), Central Finland Biobank 1–2017 and Terveystalo Biobank STB 2018001.2.

### Genotyping and quality control

Genotyping in the FinnGen cohort was performed by using Illumina (Illumina Inc., San Diego, CA, USA) and Affymetrix arrays (Thermo Fisher Scientific, Santa Clara, CA, USA) and lifted over to build version 38 (GRCh38/hg38) (52). As a sample-level quality control, individuals with high genotype absence (>5%), inexplicit sex or excess heterozygosity (±4 standard deviations) were excluded from the data (52). Additionally, in the variant level quality control, variants that had high absence (>2%), low minor allele count (<3) or low Hardy–Weinberg Equilibrium (HWE) (*P* < 1e−06) were removed [52]. A more detailed explanation of the genotyping, quality control and the genotype imputation with SiSu v3 reference panel is described in Kurki *et al*. (preprint) (52). All individuals in the cohort were Finns and matched against the SiSu v3 reference panel (http://www.sisuproject.fi/).

### Phenotype definition

In the FinnGen study, the main phenotype used in our study was TB of all organs defined using ICD-10 based diagnosis codes A15-A19. Individuals defined as a case in TB of all organs endpoint had to have at least one of the following ICD-10 codes or their subcode: A15, A16, A17, A18 or A19. Other phenotypes used were respiratory TB and TB of other organs. An individual was defined as a case in respiratory TB when that person had at least one of the following ICD-10 codes or their subcode: A15 or A16. Controls with other TB-related ICD-10 codes were excluded for respiratory TB analyses. An individual was defined as a case in TB of other organs when that person had the ICD-code A18 or one of its subcodes. Controls with other TB-related ICD-10 codes were excluded for TB of other organs analyses.

### UKB phenotype definition

From the UKB data, we obtained both self-reported and electronic health record data for disease definitions. To define the phenotypes, we used data from the self-report non-cancer illness codes (data field 20002), which were assessed during the baseline interview, hospital inpatient records (HES; data field 41234) and primary care diagnosis records (data field 42040). For TB of all organs, code 1440 was used from the self-reported data. From the hospital inpatient data, we included individuals as a case for the phenotype if they had at least one of the ICD-10 diagnosis codes used for FinnGen (see above) and, as with FinnGen, included participants with subcodes in the endpoint. Additionally, in the UKB, ICD-9 diagnosis codes 0130, 01199, 01789, 0172, 0160 and 015 were used. In the primary care data, diagnoses are coded using the NHS-specific Read v2 or CTV3 codes instead of the ICD coding. We used the following Read codes to define the respective phenotype:

- Read v2: ‘A11..’, ‘A1…’, ‘1231.’, ‘A1z..’, ‘1411.’, ‘A11z.’, ‘A11y.’, ‘A172.’, ‘A1701’, ‘A16..’, ‘A160.’, ‘A151.’, ‘A1252’, ‘65Y1.’, ‘Ayu13’, ‘Ayu1.’, ‘A1y..’, ‘A1800’, ‘A171.’, ‘A168.’, ‘A1652’, ‘A161.’, ‘A15z.’ and ‘A152.’
- CTV3: ‘A1...’, ‘A11..’, ‘1231.’, ‘1411.’, ‘X70Gy’, ‘A1z..’, ‘A11z.’, ‘A160’, ‘A151’, ‘A1y..’, ‘Xa9Cj’, ‘A152’, ‘XE0Qr’, ‘A18..’, ‘A153.’, ‘A13..’, ‘A122.’, ‘A110.’, ‘A1703’, ‘A1661’, ‘A16..’, ‘A1240’, ‘A123.’, ‘A121.’, ‘XE2aj’, ‘X70H3’, ‘Q4024’, ‘N306.’, ‘N3053’, ‘N3042’, ‘N3040’, ‘A17z.’, ‘A17y3’, ‘A17..’, ‘A1702’, ‘A15..’, ‘A14z.’, ‘A14y3’, ‘A14y1’, ‘A14..’, ‘A1252’, ‘A1243’, ‘A1241’, ‘A121z’, ‘A1210’, ‘A12..’, ‘A120.’, ‘A11y.’, ‘A111.’, ‘A101.’, ‘A1247’, ‘A1210’, ‘A12..’ or ‘A111.’

Furthermore, we excluded the following codes from controls due to TB exposure, history of personal TB or TB contact:

- ICD10-codes: B909, B908, Z201 and Z030
- Read v2: ‘ZV12A’, ‘65P2.’, ‘14P9.’, ‘ZV12B’ or ‘65 V9.’
- CTV3: ‘XaBE4’, ‘65P2.’, ‘XaCL6’, ‘XSCGJ’, ‘65 V9.’, ‘ZV011’, ‘XaK3y’, ‘X73T7’ and ‘A171z’

With this definition for TB of all organs, we ended up with 3431 cases and 442 492 controls of European ancestry. Most of the cases for TB of all organs came from the self-reported data (*N* = 1985 (57.9%)) and primary care data (N = 1298 (37.8%)).

For respiratory TB, the same ICD-10 codes were used as in FinnGen for the equivalent phenotype (see above). From the primary care data, the following Read codes were used for respiratory TB:

- Read v2: ‘A11..’, ‘A11z.’, ‘A11y.’, ‘A1252’ and ‘Ayu13’
- CTV3: ‘A11..’, ‘A11z.’, ‘A122.’, ‘A110.’, ‘A1240’, ‘A1252’, ‘A1243’, ‘A1241’, ‘A11y.’, ‘A111.’, ‘A101.’, ‘A1247’ and ‘A12..’

In addition, we excluded the following codes from controls due to other TB diagnosis, TB exposure, history of personal TB or TB contact:

- Self-reported non-cancer illness (data field 20002): 1440
- ICD-10: A17-A19, B909, B908, Z201, Z030, M900, M9000, M4909 and M4902
- ICD-9: 0130, 01199, 01789, 0172, 0160 and 015
- Read v2: ‘ZV12A’, ‘65P2.’, ‘14P9.’, ‘ZV12B’, ‘65 V9.’, ‘A1…’, ‘1231.’, ‘A1z..’, ‘1411.’, ‘A172.’, ‘A1701’, ‘A16..’, ‘A160.’, ‘A151.’, ‘65Y1.’, ‘Ayu1.’, ‘A1y..’, ‘A1800’, ‘A171.’, ‘A168.’, ‘A1652’, ‘A161.’, ‘A15z.’ and ‘A152.’
- CTV3: ‘XaBE4’, ‘65P2.’, ‘XaCL6’, ‘XSCGJ’, ‘65 V9.’, ‘ZV011’, ‘XaK3y’, ‘X73T7’, ‘A171z’, ‘A1...’, ‘1231.’, ‘1411.’, ‘X70Gy’, ‘A1z..’, ‘A160.’, ‘A151.’, ‘A1y..’, ‘Xa9Cj’, ‘A152’, ‘XE0Qr’, ‘A18..’, ‘A153.’, ‘A13..’, ‘A1703’, ‘A1661’, ‘A16..’, ‘A123.’, ‘A121.’, ‘XE2aj’, ‘X70H3’, ‘Q4024’, ‘N306.’, ‘N3053’, ‘N3042’, ‘N3040’, ‘A17z.’, ‘A17y3’, ‘A17..’, ‘A1702’, ‘A15..’, ‘A14z.’, ‘A14y3’, ‘A14y1’, ‘A14..’, ‘A121z’, ‘A1210’, ‘A12..’, ‘A120.’, ‘A1210’ and ‘A111.’

With this definition for respiratory TB, we identified 641 cases and 442 492 controls of European ancestry.

### B‌BJ phenotype definition

In the publicly available BBJ summary statistics, only respiratory TB (defined by BBJ as pulmonary TB) was available in the pre-defined endpoints of BBJ (24). Endpoints were defined using clinical data and disease records (24). For controls, samples of the cohort without the given diagnosis for respiratory TB or related codes were used (24). With this definition, the BBJ respiratory TB phenotype consisted of 7800 cases and 170 871 controls.

### HLA imputation

HLA imputation was performed for *HLA A*, *HLA B*, *HLA C*, *HLA DRB1*, *HLA DQA1*, *HLA DQB1*, *HLA DPB1*, *HLA DRB3*, *HLA DRB4* and *HLA DRB5* using HIBAG (HLA genotype imputation with attribute bagging), as implemented earlier (53).

### Statistical tools

For the FinnGen cohort, GWAS was conducted using the REGENIE pipeline (https://github.com/FINNGEN/regenie-pipelines). Manhattan-plots for FinnGen R7 GWASs and meta-analyses were plotted using R version 4.0.1 (packages: qqman and RColorBrewer). Meta-analyses were conducted using METAL (https://genome.sph.umich.edu/wiki/METAL_Documentation). Both sample size based meta-analysis and effect estimate based analyses were run. Meta-analyses were conducted for respiratory TB to achieve as harmonized phenotype between cohorts as possible. Recessive gene action test for *TYK2* P1104A variant was conducted using PLINK 1.9 (https://www.cog-genomics.org/plink/).

The UKB GWA analyses were performed using REGENIE v3.1.1 (54). The whole-genome regression model (step 1) was created using 524 307 high-quality genotyped SNPs bi-allelic; minor allele frequency (MAF) ≥ 1%; HWE *P* > 1 × 10^–6^; present in all genotype batches, total missingness <1.5% and not in a region of long-range LD (55) with the leave-one-chromosome-out (—loocv) option enabled. We corrected for the following covariates:

- age at follow-up end (August 18, 2019) or death (if earlier than follow-up end), calculated as the difference in years between the 15th day of month and year of birth (data fields 52 and 34, respectively) and the follow-up end or death date.
- sex (data field 31)
- genotyping array (categorical), derived from genotyping batch (data field 20000), as ‘UKB BiLEVE’ (−11 to −1), ‘UKB Axiom release 1’ (1 to 22) and ‘UKB Axiom release 2’ (23 to 95).
- genetic principal components 1 to 10 (data field 22009)
- centre of baseline visit (categorical; data field 54)

The GWA (step 2) was performed using v3 imputed genotypes (50) for chromosomes 1–22 and X with the approximate Firth correction applied for variants with association *P*-value <0.05 (default setting), using the flags —firth, —approx and —firth-se. After analysis with REGENIE, we excluded results for imputed variants with MAF <0.1% and/or imputation INFO<0.3.

Association testing between individual HLA alleles and TB was conducted with multivariate logistic regression using R (version 4.0.3, packages: data.table, dplyr and tidyverse). Multivariate logistic regression model was adjusted for age at death or end of follow-up (December 31, 2019), sex and the first 20 genetic principal components (adjusting for principal components accounts for population structure within the cohort). Stepwise logistic regression was conducted by adding the most strongly associated HLA allele as a covariate to the multivariate regression analysis. This was repeated as many times as there were significant alleles left in the analysis.

To assess genetic correlation between TB and different traits, we performed LD score regression analyses with LD HUB provided by the Broad Institute of Massachusetts Institute of Technology (MIT) and Harvard and MRC Integrative Epidemiology Unit, University of Bristol (56–58). Additionally, we tested AUD using LD score regression separately since the trait was not available in the LD HUB tool (56). For the LD score regression, we used the HapMap 3 SNP list and European LD score files provided with the software. Summary statistics for LD score regression were obtained from FinnGen R7 GWAS of TB of all organs and from Sanchez-Roige *et al*. for AUD (59). LD between the lead variants in the HLA region was estimated using LDpair Tool from LDlink (60).

We obtained the lead SNPs associated with smoking and used as exposure instruments against the FinnGen R7 TB GWAS summary statistics as ‘Cigarettes per day’ and ‘Age of initiation of regular smoking’ from a recent large-scale GWAS (61). The lead SNPs associated with vitamin D deficiency were obtained from the study by Revez *et al*. (62). The BMI associated SNPs and COPD associated SNPs were obtained from the Integrative Epidemiology Unit open GWAS project ID: ukb-b-19 953. AUD associated SNPs were obtained from the study by Sanchez-Roige *et al*. (59). The MR was performed using the TwoSampleMR R package (63,64). Furthermore, we tested for potential pleiotropic effects using the Egger intercept methods as part of the TwoSampleMR package and the MR-PRESSO package (65).

### Epidemiological measures

Association testing between selected known risk factors and TB was conducted with multivariate logistic regression using R (version 4.0.3, packages: data.table, dplyr and tidyverse). Multivariate logistic regression model was adjusted with age at death or end of follow-up (December 31, 2019), BMI, sex and the first 10 genetic principal components. Phenotypic traits used were as follows: smoking (ever versus never), smoking (current versus former/never), diabetes (ICD-10 codes E10-E14 or Anatomical Therapeutic Chemical [ATC]-code (medicine purchases) A10B), COPD (ICD10-code J44), hypertension (ICD10-codes I10-I15 or I67.4, or Finnish Social Insurance Institution (KELA) code 205), asthma (ICD10-code J45), CHD (ICD10-codes I20.0, I21 or I22), HIV (ICD10-codes B20-B24), RA (ICD10-codes M05 or M06), biological medication for RA (ATC-codes: L04AA24, L04AC03, L04AB04, L04AB01, L04AB06, L04AB02, L04AB05, L04AA26, L01XC02, L04AC10, L04AC07 or L04AC05), sleep apnea (ICD10-code G47.3), alcohol dependence (ICD10-code F10.2), AUD (ICD10-codes F10.1 or F10.2), inflammatory bowel disease (ICD10-codes K50, K51 or KELA codes 208 or 209) and Crohn’s disease (ICD10-code K50). Other known comorbidities such as vitamin D deficiency, organ transplantation and immunodeficiency were not included in the analyses due to the small number of cases in the FinnGen study.

A Kaplan–Meier estimator (66) was used to create survival curves representing effect of selected comorbidity to survival among TB patients. A Cox proportional hazards model (Cox regression) was used to estimate the effect of selected risk factors among TB patients on survival (67). Cox regression was adjusted with stratified sex, stratified cohort (cohort representing for example biobank or study included within the FinnGen study), BMI and the first 10 genetic principal components. Survival function for the Kaplan–Meier estimator and Cox regression was constructed using age at death or end of follow-up (December 31, 2019) as time variable and death (0 or 1) as event variable. Additionally, proportional hazards assumption of Cox regression model was tested (68). Analyses were conducted using R version 4.0.3 (packages: survival, survminer, survMisc, ggsurvplot and ggplot2).

## Funding

Business Finland (HUS 4685/31/2016 and UH4386/31/2016); AbbVie Inc.; AstraZeneca UK Ltd.; BiogenMA Inc.; Bristol Myers Squibb; Genentech Inc.; Merck Sharp Dohme Corp; Pfizer Inc.; Glaxo-SmithKline Intellectual Property Development Ltd; Sanofi US Services Inc.; Maze TherapeuticsInc.; Janssen Biotech Inc; Novartis Pharma AG and Boehringer Ingelheim. Following biobanks are acknowledged for delivering biobank samples to FinnGen: Auria Biobank (www.auria.fi/biopankki), THL Biobank (www.thl.fi/biobank), Helsinki Biobank (www.helsinginbiopankki.fi), Biobank Borealis of Northern Finland (https://www.ppshp.fi/Tutkimus-ja-opetus/Biopankki/Pages/Biobank-Borealis-briefly-in-English.aspx), Finnish Clinical Biobank Tampere (www.tays.fi/en-US/Research/and/development/Finnish/Clinical/Biobank/Tampere), Biobank of Eastern Finland (www.ita-suomenbiopankki.fi/en), Central Finland Biobank (www.ksshp.fi/fi-FI/Potilaalle/Biopankki), Finnish Red Cross Blood Service Biobank (www.veripalvelu.fi/verenluovutus/biopankkitoiminta) and Terveystalo Biobank (www.terveystalo.com/fi/Yritystietoa/Terveystalo-Biopankki/Biopankki/). All Finnish biobanks are members of BBMRI.fi infrastructure (www.bbmri.fi), and FINBB Biobank Cooperative (https://finbb.fi/) is the coordinator of the BBMRI-ERIC operations in Finland covering all Finnish biobanks.

## Data and Code Availability

Data and code used in this study are available upon reasonable request. The FinnGen individual level data may be accessed through applications to the Finnish Biobanks’ FinnBB portal, Fingenious (www.finbb.fi). Summary data can be accessed through the FinnGen site https://www.finngen.fi/en/access_results.

## Conflict of Interest statement

None declared.

## Supplementary Material

Supplemental_Information_HMG_Tervi_ddac212

Supplement_Table_S11_Tervi_et_al_ddac212

Supplement_Table_S12_Tervi_et_al_ddac212
