# Supplementary material for: Large registry-based analysis of genetic predisposition to tuberculosis identifies genetic risk factors at HLA
Source: Hum Mol Genet. 2022 Aug 26;32(1):161–71. doi: 10.1093/hmg/ddac212 (PMC9838093; doi:10.1093/hmg/ddac212)
Supplement: Supplemental_Information_HMG_Tervi_ddac212 [file supplemental_information_hmg_tervi_ddac212.pdf]

Supplements

Table of Contents

Supplemental Figures

Figure S1 – Locus zoom plot, tuberculosis all organs (*C6orf10*)

Figure S2 – Locus zoom plot, tuberculosis all organs (*INPP5A*)

Figure S3 – Manhattan plot, respiratory tuberculosis FinnGen R7

Figure S4 – Manhattan plot, tuberculosis of other organs FinnGen R7

Figure S5 – Manhattan plot, respiratory tuberculosis sample size meta-analysis

Figure S6 – Manhattan plot, respiratory tuberculosis effect estimate meta-analysis

Figure S7 – Kaplan-Meier estimate, smoking

Figure S8 – Kaplan-Meier estimate, COPD

Figure S9 – Kaplan-Meier estimate, smoking among tuberculosis cases and controls

Figure S10 – Kaplan-Meier estimate, alcohol dependence

Figure S11 – Kaplan-Meier estimate, AUD

Supplemental Tables

Table S1 – Top 20 hits for respiratory tuberculosis, sample size meta-analysis and the lead variant rs9391858

Table S2 – Top 20 hits for respiratory tuberculosis effect estimate meta-analysis and the lead variant rs9391858

Table S3 – Lead HLA-allele frequencies in FinnGen R7

Table S4 – HLA fine mapping, tuberculosis all organs

Table S5 – rs9391858 adjusted HLA fine mapping, tuberculosis all organs

Table S6 – HLA fine mapping, respiratory tuberculosis

Table S7 – Epidemiological correlates

Table S8 – Cox proportional hazards model

Table S9 – Changes of smoking over the decades

Table S10 – Genetic Correlation TB vs. AUD

Supplemental Information

Previous HLA findings from the literature

Contributors of FinnGen

Large Registry Based Analysis of Genetic Predisposition to Tuberculosis
Identifies Genetic Risk Factors at HLA
Supplements

Supplemental Figures

Figure S1 – Locus zoom plot, tuberculosis all organs (*C6orf10*)

Locus zoom plot for FinnGen R7, Tuberculosis all organs (chromosome 6, from 32.20 to 32.60).

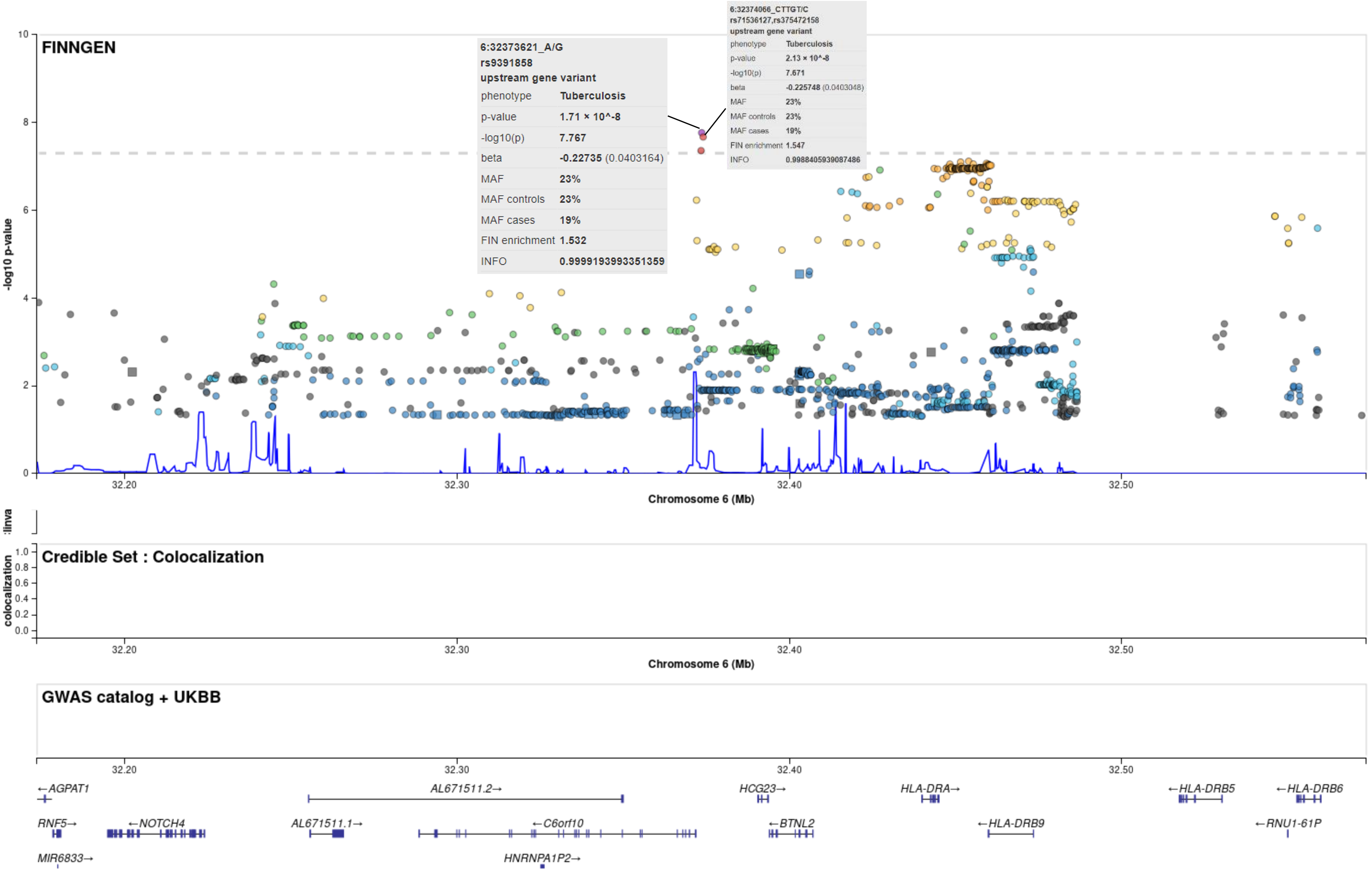

Large Registry Based Analysis of Genetic Predisposition to Tuberculosis
Identifies Genetic Risk Factors at HLA
Supplements

Figure S2 – Locus zoom plot, tuberculosis all organs (*INPP5A*)

Locus zoom plot for FinnGen R7, Tuberculosis all organs (chromosome 10, from 132.50 to 132.90).

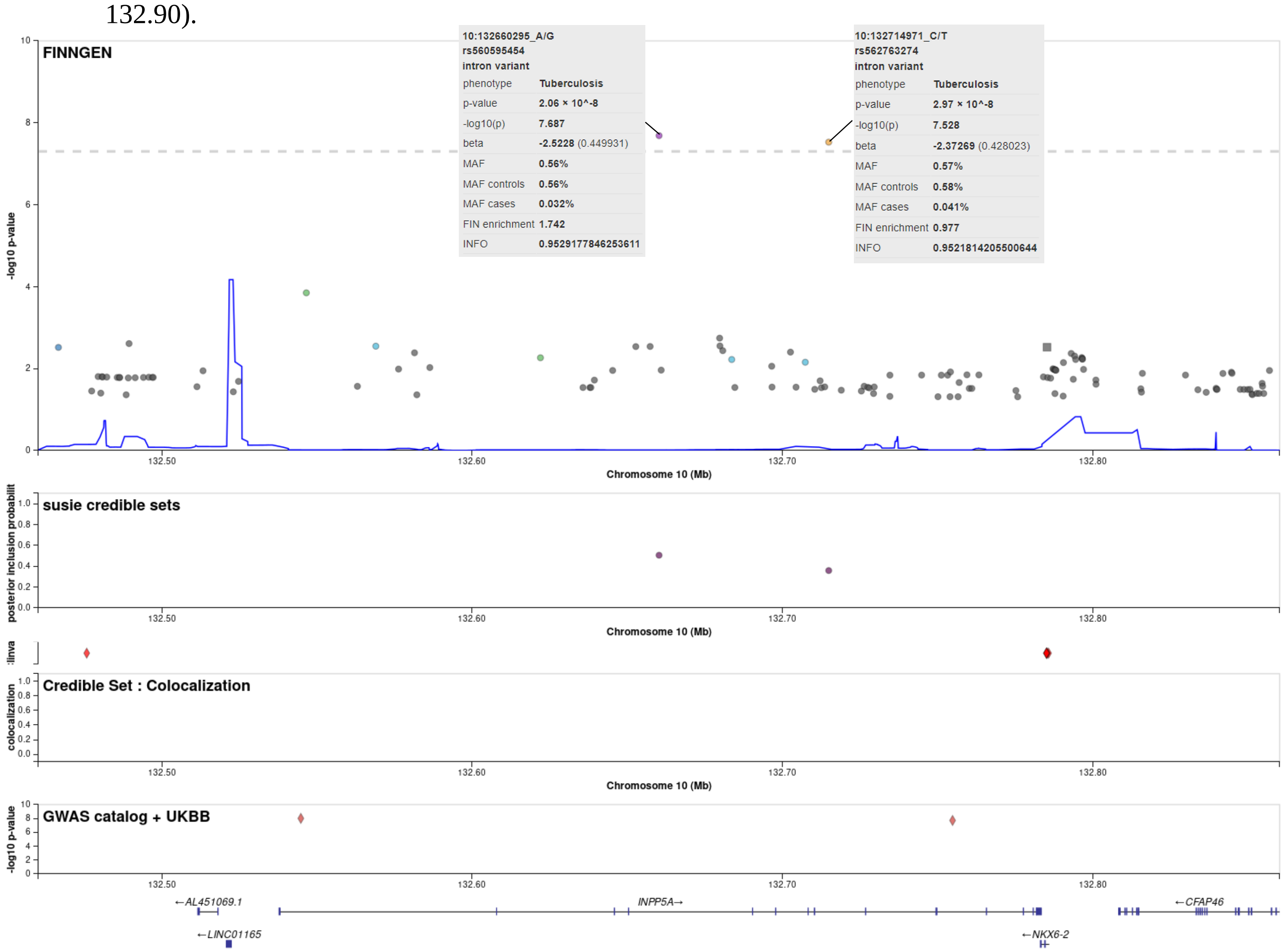

Large Registry Based Analysis of Genetic Predisposition to Tuberculosis
Identifies Genetic Risk Factors at HLA
Supplements

Figure S3 – Manhattan plot, respiratory tuberculosis FinnGen R7

Manhattan plot from FinnGen R7 GWAS – Respiratory tuberculosis (ICD-10 A15 – A16).

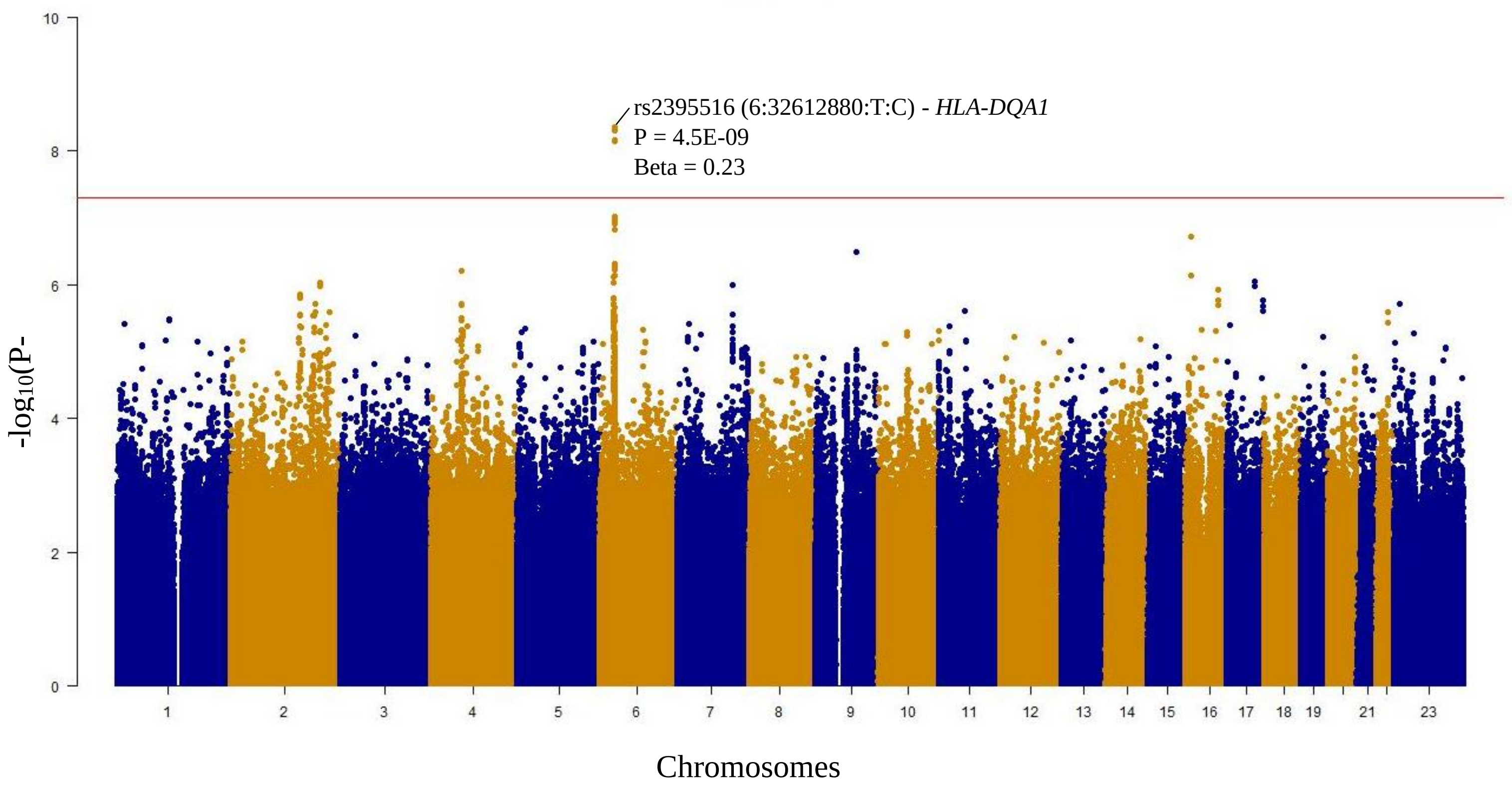

Large Registry Based Analysis of Genetic Predisposition to Tuberculosis
Identifies Genetic Risk Factors at HLA
Supplements

Figure S4 – Manhattan plot, tuberculosis of other organs FinnGen R7

Manhattan plot from FinnGen R7 GWAS – Tuberculosis of other organs (ICD-10 A18).

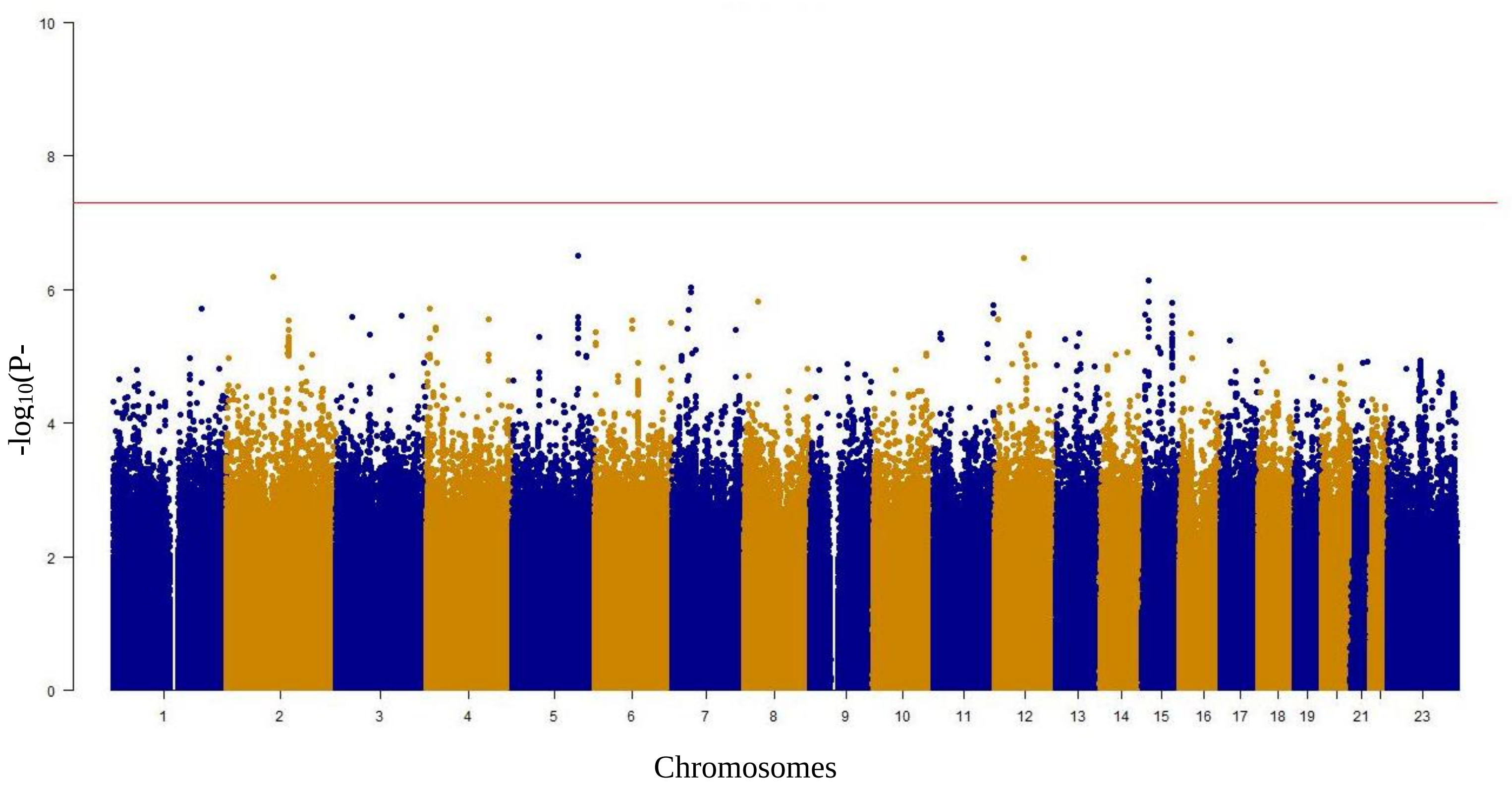

Large Registry Based Analysis of Genetic Predisposition to Tuberculosis
Identifies Genetic Risk Factors at HLA
Supplements

Figure S5 – Manhattan plot, respiratory tuberculosis sample size meta-analysis

Manhattan plot from FinnGen R7, UK Biobank and Biobank Japan, sample size meta-analysis – Respiratory tuberculosis phenotype

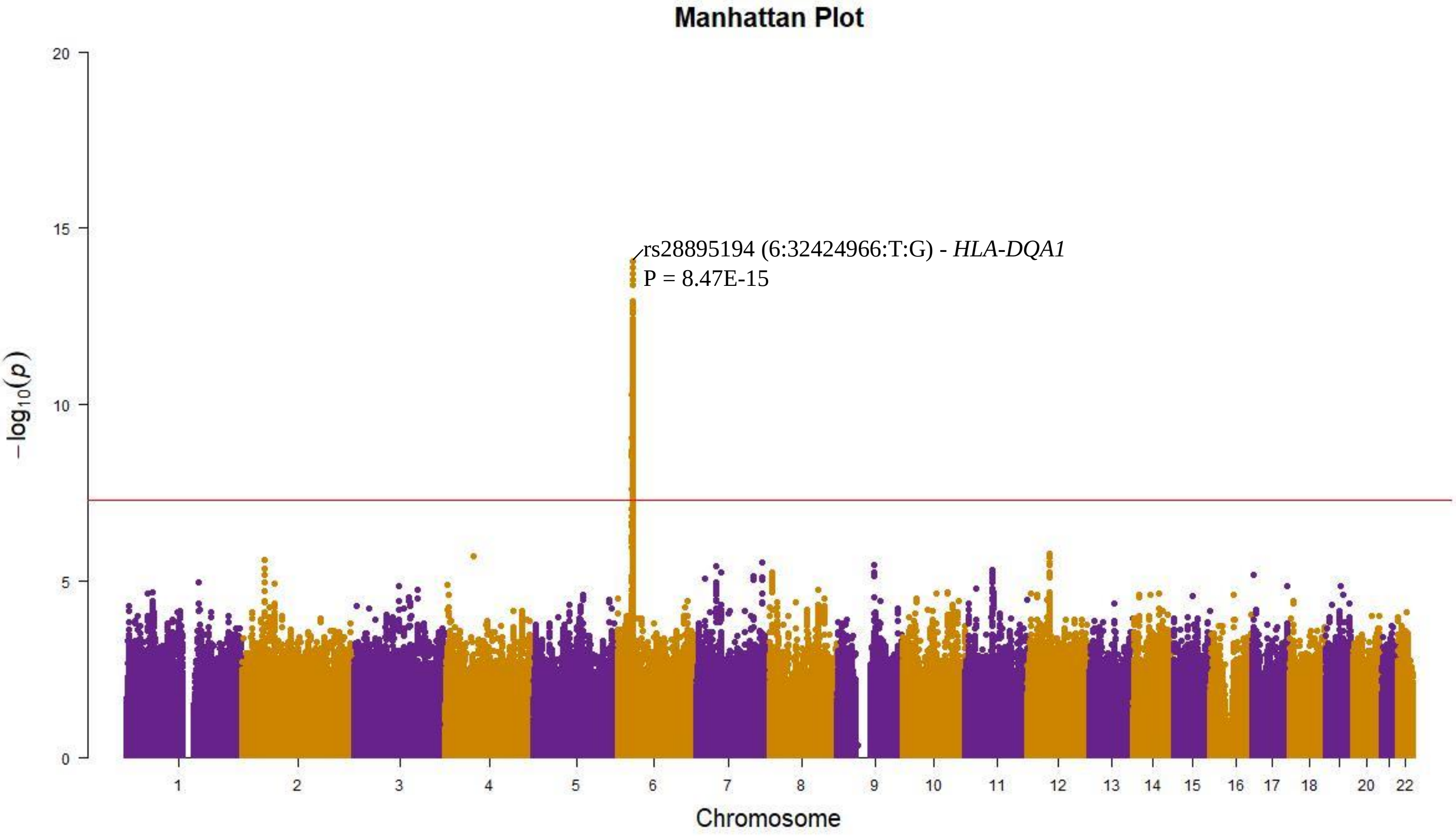

Figure S6 – Manhattan plot, respiratory tuberculosis effect estimate meta-analysis

Manhattan Plot from FinnGen R7, UK Biobank and Biobank Japan, effect estimate meta-analysis – Respiratory tuberculosis phenotype

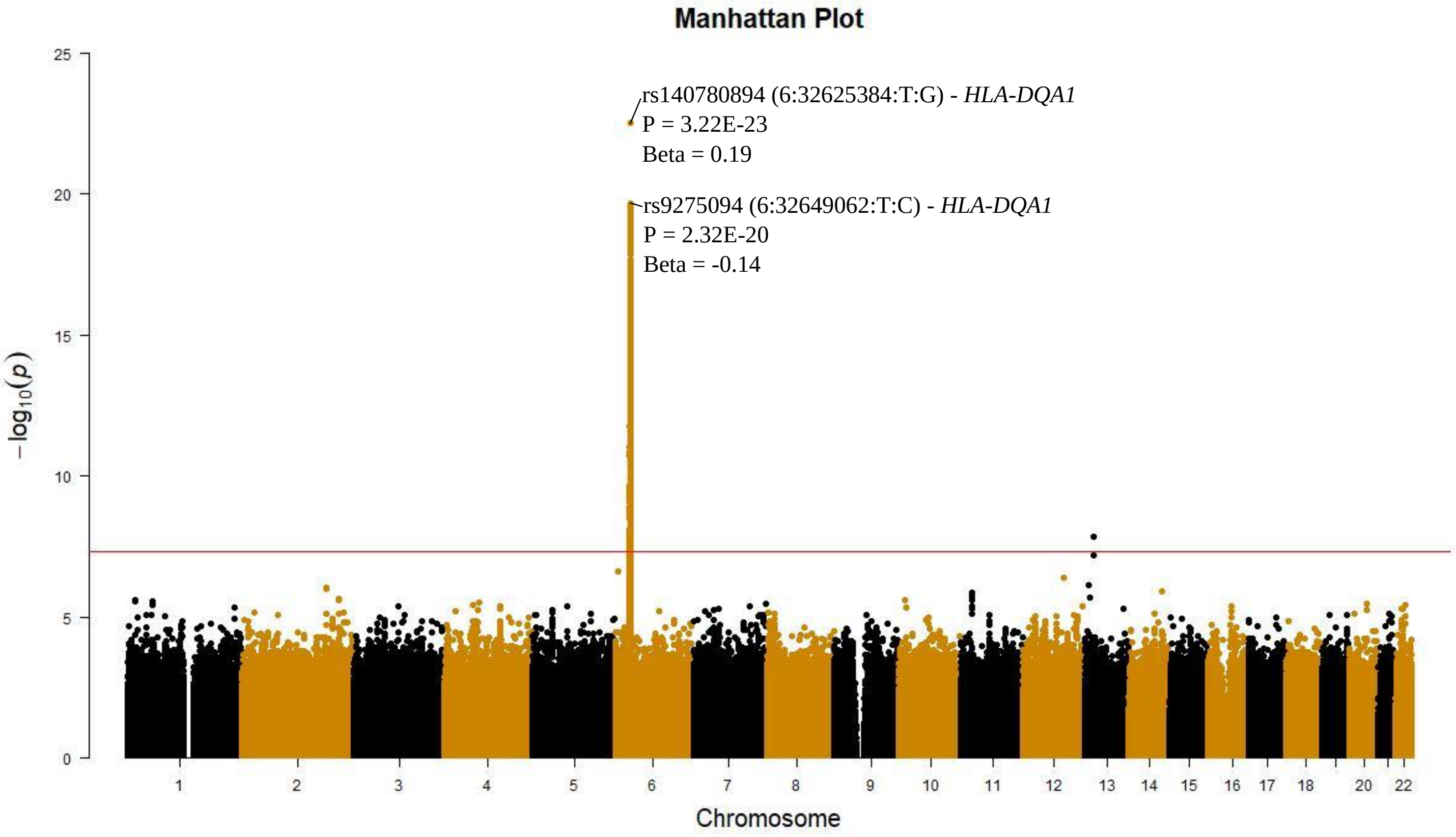

Figure S7 – Kaplan-Meier estimate, smoking

A Kaplan-Meier estimate for smoking (current, former, never) among tuberculosis cases in FinnGen R7.

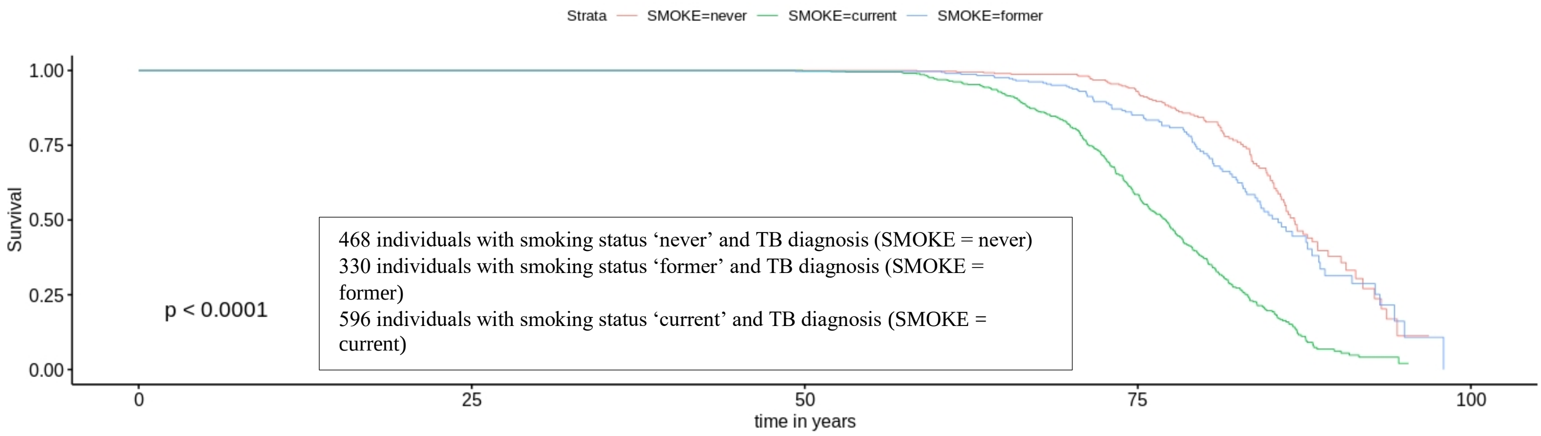

Figure S8 – Kaplan-Meier estimate, COPD

A Kaplan-Meier estimate for COPD among tuberculosis cases in FinnGen R7.

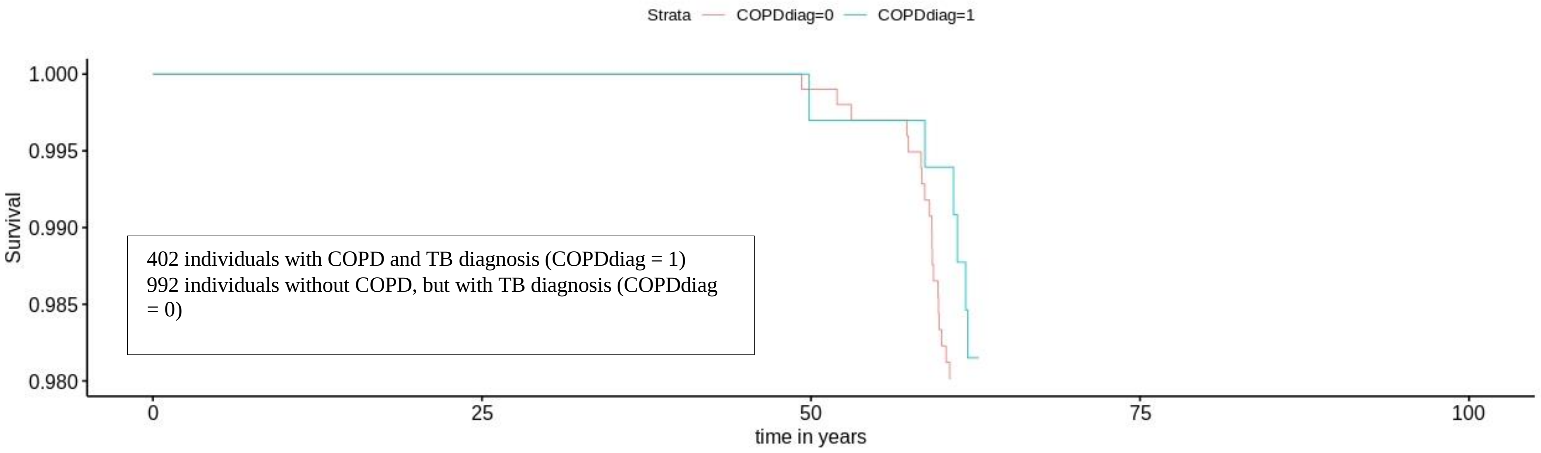

Figure S9 – Kaplan-Meier estimate, smoking among tuberculosis cases and controls

A Kaplan-Meier estimate for smoking among tuberculosis cases and controls in FinnGen R7.

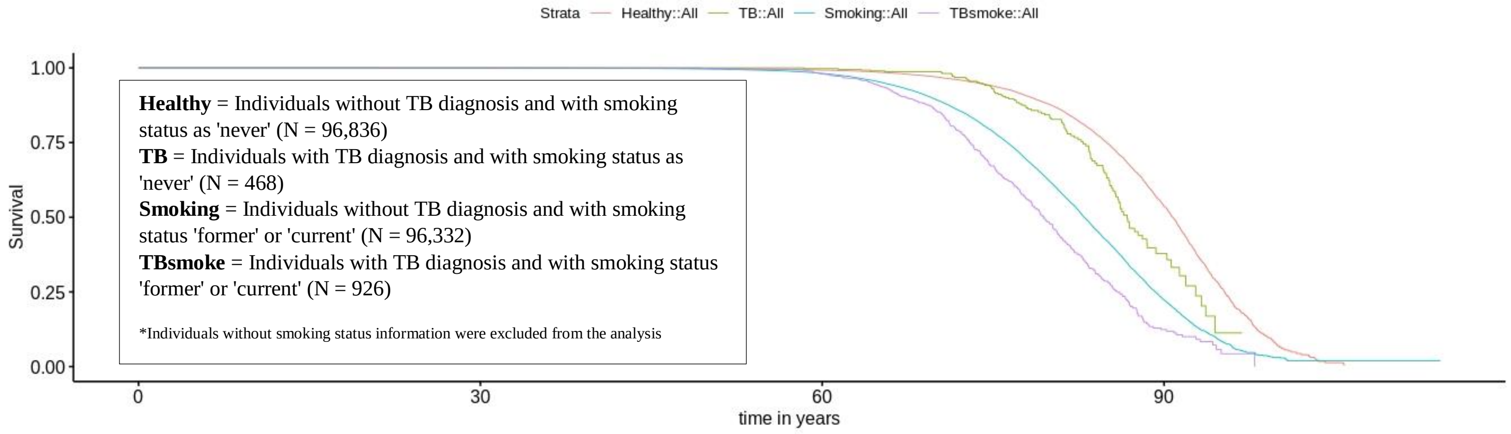

Figure S10 – Kaplan-Meier estimate, alcohol dependence

A Kaplan-Meier estimate for alcohol dependence among tuberculosis cases in FinnGen R7.

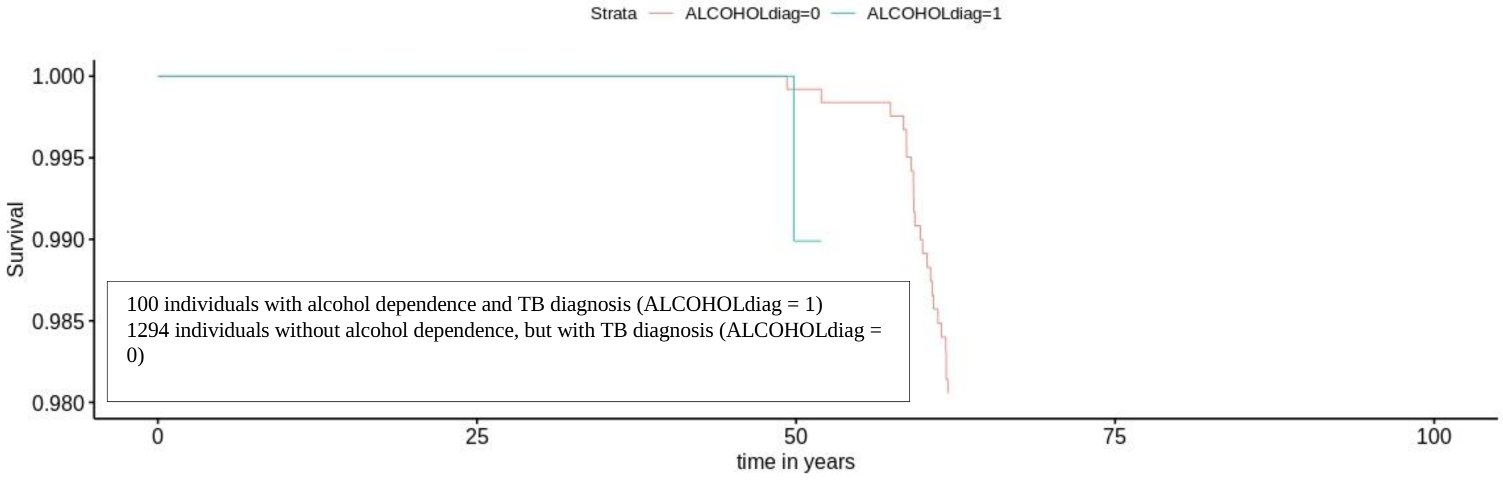

Figure S11 – Kaplan-Meier estimate, AUD

A Kaplan-Meier estimate for alcohol use disorder (AUD) among tuberculosis cases in FinnGen R7.

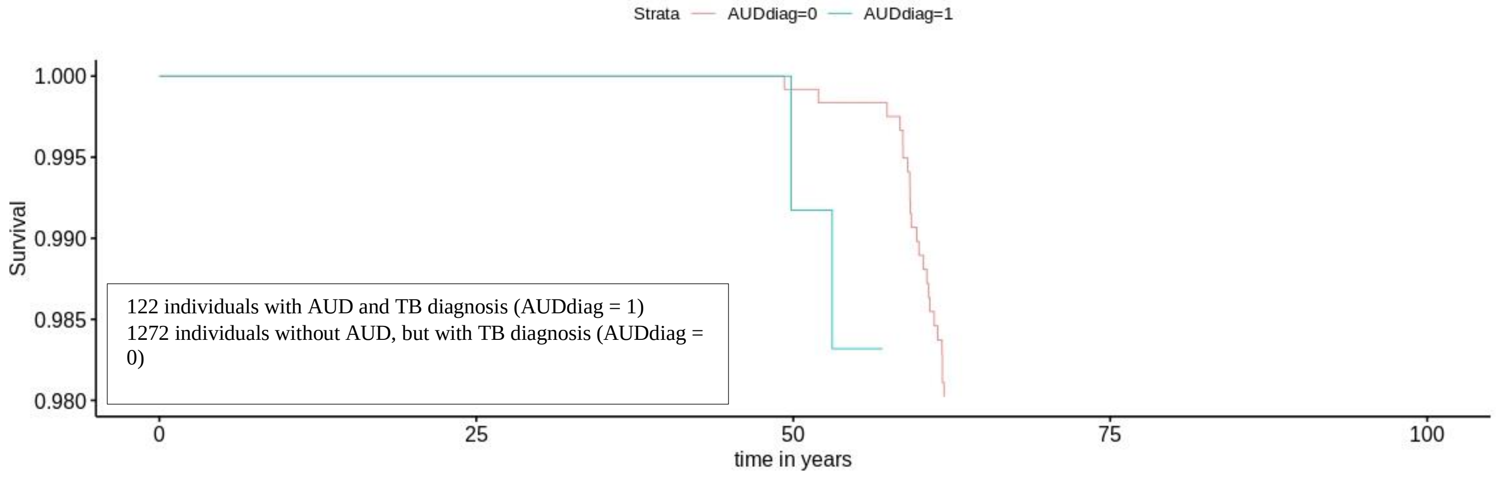

Large Registry Based Analysis of Genetic Predisposition to Tuberculosis
Identifies Genetic Risk Factors at HLA
Supplements

Supplemental Tables

Table S1 – Top 20 hits for respiratory tuberculosis, sample size meta-analysis and the lead variant rs9391858

rsID	Chromosome	Position	Alternative allele	Reference allele	N	Z-score	P-value	Direction of the effect	Hetrogeneity P-value
rs28895194	6	32424966	t	g	930098	7.76	8.47E-15	+++	0.02072
rs28895203	6	32425191	a	g	930098	-7.708	1.28E-14	---	0.01744
rs28895195	6	32424969	t	c	930098	-7.649	2.03E-14	---	0.01583
rs13194888	6	32425185	t	c	930098	-7.599	2.98E-14	---	0.01341
rs28895192	6	32424943	a	g	930098	7.563	3.94E-14	+++	0.01279
rs28895097	6	32418869	t	c	930098	-7.424	1.13E-13	---	0.00909
rs5021726	6	32578481	t	c	930098	-7.405	1.31E-13	---	7.69E-07
rs2395516	6	32580657	t	c	930098	-7.395	1.41E-13	---	4.74E-08
rs2858861	6	32580331	t	c	930098	7.393	1.43E-13	+++	9.12E-07
rs33932178	6	32584313	c	g	930098	-7.387	1.50E-13	---	5.27E-08
rs13207945	6	32579708	t	c	930098	-7.381	1.57E-13	---	8.58E-07
rs3129747	6	32581008	t	c	930098	-7.38	1.59E-13	---	6.53E-08
rs502803	6	32578958	a	g	930098	7.377	1.62E-13	+++	9.31E-07
rs532098	6	32578052	a	g	930098	7.376	1.64E-13	+++	9.13E-07
rs3104419	6	32580136	t	c	930098	7.375	1.64E-13	+++	9.31E-07
rs33915496	6	32583979	a	g	930098	-7.374	1.65E-13	---	4.75E-08
rs12523808	6	32579775	a	t	930098	-7.374	1.65E-13	---	9.22E-07
rs4959105	6	32583146	t	c	930098	7.365	1.77E-13	+++	3.91E-08
rs3104418	6	32581021	a	g	930098	7.36	1.83E-13	+++	7.87E-08
rs3104417	6	32581022	a	t	930098	7.36	1.83E-13	+++	7.87E-08
rs28895194	6	32424966	t	g	930098	7.76	8.47E-15	+++	0.02072
rs9391858	6	32341398	a	g	930098	6.13	8.81E-10	+++	0.07874

Table S2 – Top 20 hits for respiratory tuberculosis effect estimate meta-analysis and the lead variant rs9391858

**rsID**	**Chromosome**	**Position**	**Alternative allele**	**Reference allele**	**Effect estimate**	**Standard Error**	**P-value**	**Direction of the effect**	**Hetrogeneity P-value**
rs140780894	6	32625384	t	g	0.185	0.019	3.22E-23	+?+	0.3958
rs9275094	6	32649062	t	c	-0.141	0.015	2.32E-20	---	0.9697
rs9275093	6	32649047	t	c	0.141	0.015	2.35E-20	+++	0.9687
rs3021062	6	32651741	t	c	0.141	0.015	2.37E-20	+++	0.9566
rs3129785	6	32656724	t	c	0.140	0.015	2.72E-20	+++	0.9577
rs9275090	6	32648971	t	g	-0.141	0.015	2.74E-20	---	0.9592
rs9275066	6	32648404	t	c	0.141	0.015	2.87E-20	+++	0.9789
rs9275067	6	32648414	c	g	0.141	0.015	3.02E-20	+++	0.9700
rs9275096	6	32649096	t	c	0.141	0.015	3.15E-20	+++	0.9640
rs9275064	6	32648381	t	c	0.142	0.015	3.34E-20	+++	0.9729
rs3021060	6	32651846	c	g	0.141	0.015	3.36E-20	+++	0.9583
rs3021061	6	32651839	c	g	-0.141	0.015	3.36E-20	---	0.9582
rs9274791	6	32639163	t	c	0.142	0.015	3.39E-20	+++	0.9688
rs9275104	6	32649388	t	c	-0.140	0.015	3.55E-20	---	0.9620
rs9275137	6	32650880	c	g	0.140	0.015	4.04E-20	+++	0.9575
rs9275108	6	32649609	a	g	0.140	0.015	4.28E-20	+++	0.9605
rs2856677	6	32656815	t	c	0.139	0.015	4.39E-20	+++	0.9475
rs9275107	6	32649508	a	g	-0.140	0.015	4.65E-20	---	0.9605
rs9275080	6	32648627	t	c	0.140	0.015	4.77E-20	+++	0.9697
rs9275079	6	32648614	a	c	-0.140	0.015	5.01E-20	---	0.9695
rs9391858	6	32341398	a	g	0.145	0.024	1.64E-09	+++	0.0431

Table S3 – Lead HLA-allele frequencies in FinnGen R7

HLA allele	TB AF %	CTRL AF %
*HLA A*24:02*	10.08 %	9.51 %
*HLA B*35:01*	11.61 %	13.06 %
*HLA B*40:01*	7.23 %	6.16 %
*HLA C*04:01*	14.17 %	7.68 %
*HLA C*06:02*	5.33 %	6.31 %
*HLA DQA1*01:01*	15.94 %	18.55 %
*HLA DQA1*03:01*	13.06 %	11.97 %
*HLA DQB1*03:02*	13.11 %	12.04 %
*HLA DQB1*05:01*	16.49 %	19.41 %
*HLA DRB1*01:01*	15.65 %	18.29 %
*HLA DRB1*13:02*	4.70 %	3.49 %
*HLA DRB3*03:01*	4.51 %	3.40 %
*HLA DRB3*ng*	66.36 %	68.19 %

TB = Tuberculosis cases (ICD-10 A15-A19, all organs)
CTRL = Controls
AF = Allele frequency

NB! HLA alleles *HLA A*69:01, HLA DQA1*01:05, HLA DQB1*06:04, HLA DRB1*04:04* and *HLA DRB1*10:01* are not included in the list due to small number of carriers (< 5) of these alleles either in cases or in controls.

Table S4 – HLA fine mapping, tuberculosis all organs

HLA fine mapping results for tuberculosis all organs (ICD-10 A15-A19).

**HLA allele**	**P-value**	**Beta (Effect Estimate)**	**Standard Error**
*DQB1*05.01*	0.000002	-0.211	0.044
*DRB1*01.01*	0.000010	-0.199	0.045
*DQA1*01.01*	0.000014	-0.195	0.045
*DRB1*13.02*	0.000107	0.299	0.077
*DQB1*06.04*	0.000298	0.289	0.080
*DRB3*03.01*	0.000313	0.284	0.079
*C*04.01*	0.002705	-0.141	0.047
*B*35.01*	0.004252	-0.147	0.051
*B*40.01*	0.004846	0.178	0.063
*DQA1*03.01*	0.005007	0.136	0.049
*DQB1*03.02*	0.005413	0.135	0.048
*A*69.01*	0.007392	1.031	0.385
*DRB1*04.04*	0.013817	0.182	0.074
*DRB3*ng*	0.025338	-0.077	0.035
*DQA1*01.05*	0.035533	-0.462	0.220
*C*06.02*	0.042353	-0.148	0.073
*A*24.02*	0.043889	0.110	0.054
*DRB1*10.01*	0.044924	-0.431	0.215
*C*03.04*	0.050394	0.093	0.048
*B*35.03*	0.076315	0.735	0.415
*DRB4*01.03*	0.078282	0.069	0.039
*DRB1*13.01*	0.084482	0.092	0.053
*DQA1*04.01*	0.087961	0.092	0.054
*DQB1*04.02*	0.095741	0.089	0.054
*B*55.01*	0.097515	-0.481	0.290
*DRB1*08.01*	0.102866	0.088	0.054

*DRB1*08.03*	0.105056	0.621	0.383
*A*33.03*	0.105984	0.455	0.281
*C*08*	0.106903	0.946	0.586
*DPB1*14.01*	0.118091	0.201	0.129
*A*26.01*	0.122713	-0.218	0.142
*DRB4*01.03N*	0.122787	-0.238	0.154
*A*68.01*	0.123049	-0.110	0.072
*B*37.01*	0.130384	-0.332	0.220
*DRB4*ng*	0.135393	-0.057	0.038
*C*08.02*	0.136186	-0.609	0.409
*DQB1*06.03*	0.136604	0.082	0.055
*B*35.08*	0.150016	0.403	0.280
*DQA1*06.01*	0.160466	0.581	0.414
*B*13.02*	0.170272	-0.140	0.102
*DPB1*02.02*	0.203787	-1.272	1.001
*DQA1*01.03*	0.210909	0.070	0.056
*B*39.24*	0.212570	-1.250	1.002
*DRB1*04.01*	0.221706	0.068	0.056
*A*02.05*	0.223625	0.551	0.453
*DQB1*06.02*	0.236505	-0.057	0.048
*DRB1*04.08*	0.237774	0.168	0.142
*DRB5*01.01*	0.264333	-0.054	0.048
*DRB1*15.01*	0.275515	-0.052	0.048
*DRB4*01.01*	0.278185	0.185	0.170
*DRB3*01.01*	0.298123	0.044	0.042
*B*14.02*	0.303240	-0.390	0.379
*C*01.02*	0.304237	0.064	0.062
*B*44.03*	0.316183	0.167	0.166
*B*35*	0.322977	-0.990	1.001

*DQB1*06.09*	0.330164	0.311	0.319
*DQA1*01.02*	0.331786	0.040	0.042
*DPB1*11.01*	0.336032	0.323	0.336
*DRB5*ng*	0.337059	0.045	0.047
*DQA1*02.01*	0.339918	-0.070	0.073
*A*01.01*	0.340042	-0.056	0.059
*A*29.02*	0.340816	-0.338	0.355
*DRB1*12.01*	0.348530	-0.100	0.107
*DRB1*07.01*	0.356370	-0.068	0.073
*B*27.05*	0.358228	0.055	0.059
*DPB1*16.01*	0.365831	0.190	0.210
*DPB1*10.01*	0.366243	-0.454	0.502
*C*02.02*	0.366311	0.061	0.068
*A*23.01*	0.368788	0.221	0.246
*A*02.01*	0.372185	0.031	0.034
*B*40.02*	0.373153	-0.094	0.106
*DQA1*01.04*	0.379516	0.126	0.143
*A*03*	0.391737	0.385	0.449
*DQB1*03.03*	0.398762	-0.061	0.073
*DRB1*09.01*	0.403919	-0.077	0.092
*B*44.27*	0.408207	0.150	0.182
*B*44.02*	0.417924	0.053	0.066
*C*05.01*	0.426263	0.055	0.069
*DRB1*01.02*	0.426681	0.253	0.318
*DPB1*105.01*	0.433809	-0.351	0.449
*B*13.01*	0.434883	0.003	0.004
*B*46.01*	0.434883	0.003	0.004
*C*04.06*	0.434883	0.003	0.004
*DRB1*07.03*	0.434883	0.003	0.004

*DRB5*01.02*	0.434883	0.003	0.004
*DQB1*05.02*	0.445880	0.119	0.157
*DRB1*04.03*	0.452788	-0.753	1.003
*B*39.06*	0.455167	0.378	0.506
*B*07*	0.459281	0.371	0.502
*C*15.02*	0.463271	-0.077	0.105
*A*11.01*	0.466173	-0.061	0.084
*DRB1*07*	0.469309	0.732	1.012
*DQB1*05.03*	0.480477	0.105	0.149
*C*12.02*	0.493963	-0.680	0.995
*DQA1*03*	0.500647	0.156	0.231
*B*27.02*	0.509634	0.272	0.412
*C*07.01*	0.520862	0.031	0.049
*DRB1*14.01*	0.522911	0.194	0.303
*DRB1*04.05*	0.523474	0.456	0.715
*DRB1*11.04*	0.524703	0.165	0.260
*C*14.02*	0.527574	-0.090	0.143
*B*35.02*	0.565691	-0.405	0.706
*DRB1*08.02*	0.566130	0.236	0.411
*B*57.01*	0.573940	-0.086	0.152
*DQB1*02*	0.576471	-0.561	1.005
*C*07.02*	0.580957	0.025	0.046
*A*30*	0.581169	-0.247	0.449
*DRB1*11.03*	0.582952	-0.389	0.708
*DQA1*03.03*	0.590646	0.054	0.100
*B*18.01*	0.591410	0.040	0.074
*DRB5*02.02*	0.594485	0.095	0.179
*B*52.01*	0.600122	-0.522	0.995
*C*12.03*	0.603454	0.047	0.090

*C*07.04*	0.628729	0.056	0.116
*DQA1*05.01*	0.638531	-0.026	0.055
*B*15.17*	0.639221	-0.333	0.711
*B*41.01*	0.647245	-0.163	0.356
*A*29.01*	0.648677	-0.153	0.335
*DQB1*02.01*	0.652257	-0.025	0.055
*B*58.01*	0.663105	0.118	0.270
*DRB1*03.01*	0.666015	-0.024	0.055
*C*17.01*	0.673920	-0.150	0.356
*DQA1*05.05*	0.694599	-0.025	0.064
*B*15.01*	0.701440	0.018	0.048
*DRB1*13.03*	0.705854	0.087	0.231
*DPB1*15.01*	0.712830	0.085	0.231
*C*17.03*	0.721757	0.120	0.336
*DRB1*14.54*	0.722212	0.060	0.168
*DRB1*16.01*	0.757677	0.054	0.176
*B*41.02*	0.757802	0.104	0.336
*DPB1*19.01*	0.758384	-0.075	0.244
*C*07*	0.780485	0.277	0.994
*B*14.01*	0.785600	-0.194	0.711
*C*03.02*	0.792337	0.071	0.270
*B*18*	0.797807	-0.182	0.711
*DPB1*13.01*	0.804517	-0.075	0.304
*B*47.01*	0.806018	-0.049	0.198
*A*30.01*	0.815632	-0.078	0.336
*DQA1*03.02*	0.818974	-0.019	0.082
*A*03.01*	0.820104	-0.009	0.038
*A*31.01*	0.827967	0.018	0.082
*A*32.01*	0.830886	-0.018	0.086

*DRB1*14.02*	0.836961	-0.056	0.270
*B*39.01*	0.838654	0.018	0.086
*DRB1*14*	0.839716	0.204	1.007
*DQA1*05.03*	0.853006	-0.050	0.270
*DRB1*11.01*	0.854295	0.016	0.086
*A*25.01*	0.866136	0.025	0.150
*DPB1*17.01*	0.880557	-0.039	0.261
*B*38.01*	0.886412	0.027	0.188
*B*44*	0.891921	-0.097	0.713
*B*51.01*	0.893341	0.009	0.068
*B*08.01*	0.894089	0.008	0.057
*C*03.03*	0.894630	-0.008	0.059
*DPB1*06.01*	0.896674	-10.420	80.241
*B*49.01*	0.896761	0.059	0.452
*B*39*	0.898250	-10.315	80.666
*A*33.01*	0.903346	-11.352	93.479
*B*07.02*	0.903382	0.006	0.048
*DRB1*01.03*	0.904151	-10.447	86.756
*C*04*	0.914070	-8.429	78.113
*DPB1*05.01*	0.914408	-0.012	0.109
*DRB1*15.02*	0.914653	-10.205	95.226
*DQB1*06.01*	0.917649	-10.213	98.778
*A*02.02*	0.920117	-9.309	92.825
*DPB1*20.01*	0.920342	-0.023	0.226
*A*02*	0.920844	-10.420	104.860
*B*15*	0.923330	-10.320	107.235
*B*13*	0.928320	-7.913	87.961
*B*45.01*	0.930779	0.044	0.504
*B*15.16*	0.931612	-7.111	82.865

*B*51*	0.932484	0.038	0.450
*DQB1*03.01*	0.932552	0.004	0.052
*C*12*	0.933599	-8.214	98.590
*DQB1*03.04*	0.933720	-9.309	111.932
*DQB1*06*	0.934338	-7.146	86.740
*DQB1*02.02*	0.934965	-0.007	0.083
*DQB1*04*	0.935148	-7.410	91.066
*DPB1*04.02*	0.935298	-0.003	0.042
*A*01*	0.935813	-7.450	92.511
*C*01*	0.936556	-6.209	78.007
*C*16.02*	0.940329	-7.112	95.013
*A*02.17*	0.940491	-7.768	104.051
*C*03*	0.941529	-6.314	86.080
*A*32*	0.941776	-6.278	85.961
*DPB1*04.01*	0.942379	-0.002	0.033
*DRB1*15*	0.942691	-7.101	98.775
*C*02*	0.944063	-5.833	83.133
*DPB1*02.01*	0.944613	-0.003	0.047
*A*31*	0.944703	-8.551	123.281
*DPB1*09.01*	0.946733	-7.013	104.964
*C*06*	0.947169	-5.530	83.450
*A*28*	0.947191	-6.353	95.920
*A*11*	0.948528	-8.078	125.127
*A*30.02*	0.949659	-7.163	113.460
*A*01.02*	0.951458	-7.207	118.388
*A*02.06*	0.951681	-6.621	109.266
*A*68*	0.951716	-6.721	111.004
*B*40*	0.952944	-6.594	111.746
*DRB1*13*	0.953231	-6.482	110.525

*DRB3*02.02*	0.954588	-0.003	0.054
*B*27*	0.955189	-6.118	108.872
*B*08*	0.955428	-5.396	96.542
*DPB1*03.01*	0.958495	-0.003	0.048
*DRB1*04.02*	0.958665	-4.228	81.581
*DPB1*01.01*	0.958863	0.004	0.071
*A*68167*	0.959824	-5.478	108.737
*C*15.05*	0.961949	-0.048	1.007
*A*24*	0.962440	0.033	0.706
*C*05*	0.964255	-5.354	119.468
*DPB1*34.01*	0.964308	-5.346	119.468
*B*50.01*	0.975808	-0.015	0.503
*C*16.01*	0.985346	-0.005	0.269
*B*56.01*	0.986736	0.002	0.117
*DRB1*04.07*	0.999170	0.000	0.450

Table S5 – rs9391858 adjusted HLA fine mapping, tuberculosis all organs

HLA fine mapping results for tuberculosis all organs (ICD-10 A15-A19) adjusted with the lead SNP rs9391858.

**HLA allele**	**P-value**	**Beta (Effect Estimate)**	**Standard Error**
*DRB1*13.02*	0.001	0.252	0.078
*DQB1*06.04*	0.003	0.242	0.080
*DRB3*03.01*	0.003	0.237	0.079
*A*69.01*	0.010	0.994	0.385
*DPB1*04.02*	0.013	0.112	0.045
*B*40.01*	0.020	0.148	0.063
*DQB1*06.02*	0.020	-0.115	0.049
*DRB5*01.01*	0.023	-0.112	0.049
*DRB1*15.01*	0.024	-0.110	0.049
*DRB5*ng*	0.030	0.104	0.048
*A*68.01*	0.056	-0.137	0.072
*A*24.02*	0.069	0.099	0.054
*C*06.02*	0.069	-0.132	0.073
*B*55.01*	0.073	-0.520	0.290
*DRB1*04.04*	0.075	0.133	0.074
*DQA1*03.01*	0.086	0.085	0.049
*B*35.03*	0.089	0.704	0.415
*DQB1*03.02*	0.090	0.084	0.049
*A*33.03*	0.094	0.471	0.281
*C*08.02*	0.111	-0.652	0.409
*C*03.04*	0.111	0.076	0.048
*B*13.02*	0.112	-0.162	0.102
*C*08*	0.129	0.890	0.587
*A*26.01*	0.130	-0.214	0.142
*DRB1*08.03*	0.130	0.580	0.383

*DQA1*05.01*	0.144	-0.082	0.056
*DQB1*02.01*	0.149	-0.081	0.056
*DPB1*14.01*	0.150	0.185	0.129
*DRB1*03.01*	0.155	-0.079	0.056
*DRB1*12.01*	0.158	-0.152	0.107
*A*01.01*	0.186	-0.078	0.059
*DQA1*06.01*	0.187	0.546	0.414
*DPB1*02.02*	0.192	-1.307	1.001
*B*35.08*	0.195	0.363	0.280
*B*39.24*	0.197	-1.293	1.002
*DQA1*01.05*	0.202	-0.284	0.223
*A*03.01*	0.216	0.048	0.039
*C*01.02*	0.217	0.077	0.062
*B*27.05*	0.219	0.073	0.059
*DQA1*05.05*	0.223	-0.078	0.064
*B*37.01*	0.242	-0.257	0.220
*DRB1*10.01*	0.247	-0.252	0.218
*B*14.02*	0.250	-0.436	0.379
*A*02.05*	0.257	0.513	0.453
*B*40.02*	0.289	-0.113	0.106
*DRB3*02.02*	0.290	-0.058	0.054
*B*35*	0.305	-1.028	1.001
*A*29.02*	0.309	-0.361	0.355
*DQA1*01.01*	0.339	0.089	0.093
*DQB1*03.01*	0.345	-0.050	0.053
*DPB1*10.01*	0.357	-0.463	0.502
*DPB1*04.01*	0.364	-0.031	0.034
*DRB1*07*	0.369	0.910	1.013

*C*14.02*	0.371	-0.128	0.143
*DRB1*01.01*	0.376	0.085	0.096
*DRB1*04.08*	0.386	0.123	0.142
*DPB1*11.01*	0.393	0.287	0.336
*DRB4*ng*	0.394	-0.032	0.038
*DRB4*01.01*	0.398	0.144	0.171
*A*03*	0.399	0.378	0.449
*DQB1*06.09*	0.410	0.263	0.319
*A*11.01*	0.416	-0.068	0.084
*DQA1*02.01*	0.423	-0.059	0.073
*B*13.01*	0.425	0.003	0.004
*B*46.01*	0.425	0.003	0.004
*C*04.06*	0.425	0.003	0.004
*DRB1*07.03*	0.425	0.003	0.004
*DRB5*01.02*	0.425	0.003	0.004
*B*44.03*	0.428	0.132	0.166
*DRB1*04.03*	0.429	-0.793	1.003
*DRB4*01.03*	0.433	0.031	0.040
*DPB1*03.01*	0.435	-0.038	0.049
*A*23.01*	0.436	0.192	0.246
*DRB1*07.01*	0.441	-0.057	0.073
*DRB1*13.01*	0.451	0.041	0.054
*B*08.01*	0.472	-0.041	0.057
*DQA1*04.01*	0.483	0.038	0.054
*C*02.02*	0.485	0.047	0.068
*DPB1*16.01*	0.496	0.143	0.210
*DQB1*04.02*	0.508	0.036	0.054
*C*12.02*	0.510	-0.655	0.993

*DRB1*01.02*	0.511	0.209	0.318
*B*39.06*	0.512	0.332	0.506
*C*04.01*	0.514	-0.034	0.052
*A*30*	0.519	-0.289	0.449
*B*07*	0.519	0.324	0.502
*DQB1*02.02*	0.522	-0.053	0.083
*DRB1*08.01*	0.523	0.035	0.055
*B*35.02*	0.525	-0.448	0.706
*DRB1*11.03*	0.533	-0.442	0.708
*DQB1*02*	0.544	-0.610	1.005
*DRB1*04.05*	0.565	0.412	0.715
*DQA1*01.04*	0.573	0.081	0.143
*B*44.27*	0.575	0.102	0.182
*B*27.02*	0.575	0.231	0.412
*DQB1*06.03*	0.575	0.031	0.056
*B*15.17*	0.595	-0.378	0.711
*B*52.01*	0.598	-0.524	0.994
*DPB1*01.01*	0.599	-0.038	0.071
*DRB1*14.01*	0.625	0.148	0.303
*C*03.03*	0.625	-0.029	0.059
*DQA1*03*	0.625	0.113	0.232
*DRB1*11.04*	0.647	0.119	0.260
*B*47.01*	0.649	-0.090	0.198
*DQB1*05.02*	0.658	0.069	0.157
*B*58.01*	0.666	0.116	0.270
*C*12.03*	0.671	0.038	0.090
*DRB1*08.02*	0.678	0.171	0.411
*DQA1*01.02*	0.683	-0.017	0.043

*B*51.01*	0.683	0.028	0.068
*DQB1*05.03*	0.683	0.061	0.149
*DPB1*19.01*	0.695	-0.096	0.244
*DRB1*14.02*	0.698	-0.105	0.270
*DPB1*105.01*	0.699	-0.174	0.450
*DRB1*11.01*	0.704	-0.033	0.087
*DPB1*05.01*	0.706	-0.041	0.109
*A*32.01*	0.708	-0.032	0.086
*A*30.01*	0.710	-0.125	0.336
*DQA1*05.03*	0.713	-0.099	0.270
*DRB3*01.01*	0.713	-0.016	0.043
*C*07.01*	0.715	-0.018	0.049
*DQB1*05.01*	0.731	0.034	0.099
*C*07.04*	0.735	0.039	0.116
*B*14.01*	0.741	-0.235	0.711
*B*07.02*	0.744	-0.016	0.048
*DQA1*01.03*	0.746	0.018	0.056
*DRB4*01.03N*	0.748	-0.051	0.158
*DQA1*03.02*	0.751	0.026	0.082
*C*07.02*	0.752	-0.015	0.046
*DPB1*17.01*	0.758	-0.080	0.261
*A*02.01*	0.760	0.011	0.035
*A*25.01*	0.763	0.045	0.150
*DQB1*03.03*	0.763	0.022	0.074
*DPB1*20.01*	0.770	-0.066	0.226
*C*07*	0.772	0.288	0.992
*DRB1*04.01*	0.777	0.016	0.056
*B*44.02*	0.784	0.018	0.066

*B*18*	0.790	-0.190	0.711
*B*57.01*	0.797	0.040	0.154
*DPB1*15.01*	0.802	0.058	0.231
*C*03.02*	0.805	0.066	0.270
*DRB5*02.02*	0.806	0.044	0.179
*C*17.03*	0.824	0.075	0.336
*C*05.01*	0.828	0.015	0.070
*B*56.01*	0.833	-0.025	0.117
*DRB3*ng*	0.833	-0.008	0.037
*B*39.01*	0.837	-0.018	0.087
*B*18.01*	0.839	0.015	0.074
*B*41.02*	0.859	0.060	0.336
*DRB1*13.03*	0.864	0.040	0.232
*B*15.01*	0.870	-0.008	0.048
*B*35.01*	0.877	-0.009	0.059
*DPB1*13.01*	0.882	-0.045	0.304
*C*16.01*	0.885	-0.039	0.269
*DRB1*14*	0.890	0.140	1.007
*DRB1*09.01*	0.894	-0.012	0.092
*DPB1*06.01*	0.896	-10.465	80.219
*B*39*	0.898	-10.350	80.622
*B*50.01*	0.900	-0.063	0.503
*DPB1*02.01*	0.901	-0.006	0.047
*A*33.01*	0.903	-11.386	93.360
*DRB1*01.03*	0.906	-10.269	86.757
*C*04*	0.913	-8.493	78.046
*DRB1*15.02*	0.914	-10.245	95.242
*B*49.01*	0.916	0.048	0.452

*DQB1*06.01*	0.917	-10.259	98.781
*A*02.02*	0.920	-9.348	92.682
*A*02*	0.921	-10.355	104.917
*B*15*	0.923	-10.373	107.065
*A*29.01*	0.925	-0.032	0.336
*C*15.05*	0.926	-0.093	1.007
*B*13*	0.927	-7.993	87.802
*DRB1*04.07*	0.929	-0.040	0.450
*B*15.16*	0.931	-7.177	82.471
*DRB1*14.54*	0.931	0.015	0.168
*DQB1*03.04*	0.933	-9.354	111.857
*C*12*	0.933	-8.232	98.658
*DQB1*06*	0.935	-7.143	87.420
*A*01*	0.935	-7.539	92.339
*DQB1*04*	0.935	-7.428	91.720
*C*01*	0.938	-6.107	77.907
*C*16.02*	0.941	-7.102	95.578
*A*32*	0.941	-6.375	85.965
*A*02.17*	0.942	-7.628	103.969
*C*03*	0.942	-6.239	86.321
*A*31*	0.944	-8.618	122.747
*DRB1*15*	0.944	-6.930	98.789
*C*02*	0.945	-5.687	83.136
*A*28*	0.946	-6.464	95.917
*DPB1*09.01*	0.947	-6.993	104.617
*C*06*	0.949	-5.385	83.400
*A*30.02*	0.949	-7.185	112.563
*A*11*	0.949	-7.999	125.783

*DQA1*03.03*	0.949	0.006	0.100
*A*01.02*	0.951	-7.251	117.572
*A*02.06*	0.952	-6.595	108.686
*DRB1*13*	0.952	-6.594	110.533
*A*68*	0.953	-6.532	111.157
*B*40*	0.954	-6.556	112.491
*B*08*	0.955	-5.481	96.539
*B*27*	0.956	-5.999	109.383
*DRB1*04.02*	0.958	-4.339	81.494
*A*68167*	0.960	-5.475	108.777
*C*05*	0.964	-5.445	119.468
*DPB1*34.01*	0.964	-5.445	119.468
*B*38.01*	0.969	-0.007	0.188
*C*17.01*	0.975	0.011	0.357
*DRB1*16.01*	0.976	0.005	0.176
*B*51*	0.984	-0.009	0.450
*B*44*	0.990	0.009	0.713
*B*45.01*	0.991	-0.006	0.504
*A*24*	0.992	-0.007	0.706
*A*31.01*	0.992	0.001	0.082
*C*15.02*	0.993	0.001	0.106
*B*41.01*	0.996	-0.002	0.357
**SNP (rs9391858)**	**0.425**	**0.003**	**0.004**

Table S6 – HLA fine mapping, respiratory tuberculosis

HLA fine mapping results for respiratory tuberculosis (ICD-10 A15-A16).

HLA allele	P-value	Beta (Effect Estimate)	Standard Error
*DQB1*05.01*	0.00003	-0.21753	0.05169
*DRB1*01.01*	0.00009	-0.20774	0.05288
*DQA1*01.01*	0.00016	-0.19802	0.05239
*DRB1*13.02*	0.00019	0.33327	0.08927
*DRB3*03.01*	0.00043	0.32038	0.09097
*DQB1*06.04*	0.00045	0.32421	0.09238
*DQB1*03.02*	0.00202	0.17275	0.05595
*DQA1*03.01*	0.00252	0.16963	0.05615
*DRB1*04.04*	0.00531	0.23646	0.08483
*A*69.01*	0.00604	1.14084	0.41552
*B*40.01*	0.00885	0.19298	0.07372
*C*03.04*	0.01038	0.14063	0.05487
*C*04.01*	0.01131	-0.13890	0.05484
*DQA1*04.01*	0.01941	0.14360	0.06143
*DRB1*08.01*	0.02526	0.13844	0.06187
*DQB1*04.02*	0.02544	0.13749	0.06153
*C*08*	0.03694	1.22512	0.58719
*DQA1*01.05*	0.04070	-0.55021	0.26884
*A*01.01*	0.04568	-0.14224	0.07118
*DPB1*14.01*	0.05511	0.27778	0.14483
*DRB1*10.01*	0.05594	-0.49646	0.25972
*DRB3*ng*	0.05725	-0.07708	0.04054
*B*35.01*	0.05788	-0.11221	0.05916
*B*55.01*	0.06065	-0.71166	0.37934
*B*13.02*	0.06110	-0.23111	0.12341
*DRB1*13.01*	0.08344	0.10718	0.06191

*C*06.02*	0.10229	-0.13803	0.08448
*DRB4*01.03*	0.10760	0.07343	0.04563
*B*15.01*	0.11078	0.08744	0.05483
*A*03*	0.13353	0.67332	0.44879
*DQB1*06.03*	0.13765	0.09595	0.06463
*DPB1*19.01*	0.14382	-0.51810	0.35444
*A*24.02*	0.15339	0.09170	0.06423
*C*08.02*	0.15588	-0.70956	0.50001
*B*35.08*	0.16061	0.44773	0.31912
*DRB4*ng*	0.19074	-0.05800	0.04433
*DQA1*01.03*	0.19665	0.08370	0.06482
*DRB1*01.02*	0.19740	0.43127	0.33457
*DRB1*09.01*	0.19760	-0.14297	0.11096
*DQB1*06.02*	0.20226	-0.07270	0.05701
*DRB1*15.01*	0.20982	-0.07115	0.05673
*DRB5*01.01*	0.21071	-0.07109	0.05679
*DRB5*ng*	0.22298	0.06756	0.05544
*A*33.03*	0.23852	0.39729	0.33706
*DQA1*02.01*	0.23904	-0.10235	0.08693
*DRB1*12.01*	0.23917	-0.15031	0.12770
*DRB4*01.03N*	0.23953	-0.20997	0.17852
*B*37.01*	0.24279	-0.29399	0.25169
*DRB1*07.01*	0.24971	-0.10010	0.08696
*DRB1*04.01*	0.25218	0.07417	0.06478
*DQB1*03.03*	0.27687	-0.09419	0.08662
*DRB1*04.05*	0.27833	0.77599	0.71581
*C*02.02*	0.28204	0.08426	0.07832
*DRB1*07*	0.29469	1.06202	1.01349
*B*13.01*	0.32141	0.00419	0.00422

*B*46.01*	0.32141	0.00419	0.00422
*C*04.06*	0.32141	0.00419	0.00422
*DRB1*07.03*	0.32141	0.00419	0.00422
*DRB5*01.02*	0.32141	0.00419	0.00422
*B*39.24*	0.32389	-0.98925	1.00279
*DPB1*01.01*	0.32523	-0.08473	0.08613
*C*05.01*	0.32762	0.07859	0.08028
*DQA1*01.04*	0.34942	0.15544	0.16612
*DPB1*105.01*	0.35610	-0.53404	0.57870
*DQA1*05.01*	0.36690	-0.05885	0.06523
*DRB3*01.01*	0.37147	0.04402	0.04925
*DRB1*03.01*	0.37490	-0.05797	0.06533
*C*14.02*	0.39044	-0.14848	0.17289
*DQB1*02.01*	0.39187	-0.05595	0.06534
*B*44.02*	0.39301	0.06537	0.07654
*B*51*	0.40136	0.37760	0.44996
*DRB1*04.08*	0.40583	0.14047	0.16899
*DRB1*08.02*	0.40655	0.37323	0.44968
*DRB1*04.07*	0.41675	0.36588	0.45055
*A*26.01*	0.43401	-0.12349	0.15785
*DRB1*11.04*	0.43632	-0.29533	0.37940
*DQA1*05.03*	0.43936	0.21667	0.28020
*C*03.03*	0.45145	0.05057	0.06716
*DRB1*14.02*	0.45221	0.21063	0.28018
*B*39.06*	0.45785	0.43263	0.58274
*DQB1*05.03*	0.46053	0.12789	0.17330
*DPB1*10.01*	0.46189	-0.42623	0.57932
*A*30*	0.47693	-0.41139	0.57840
*DPB1*15.01*	0.47730	-0.22543	0.31722

*DRB1*11.03*	0.47972	-0.70700	1.00035
*B*38.01*	0.48053	-0.17284	0.24500
*DQA1*05.05*	0.48820	-0.05198	0.07499
*B*35*	0.49142	-0.68843	1.00054
*DQB1*06.09*	0.49715	0.25826	0.38037
*DPB1*17.01*	0.51100	-0.22058	0.33560
*B*08.01*	0.51751	-0.04397	0.06793
*C*01.02*	0.51758	0.04751	0.07342
*B*40.02*	0.52233	-0.07884	0.12323
*A*02.05*	0.52606	0.36914	0.58222
*C*07*	0.53185	0.61718	0.98721
*DQA1*01.02*	0.53395	0.03052	0.04907
*A*23.01*	0.53898	0.17929	0.29184
*DQA1*03.02*	0.54808	-0.05862	0.09760
*B*14.02*	0.54895	-0.24492	0.40865
*DPB1*03.01*	0.56519	0.03203	0.05568
*B*14.01*	0.57106	-0.56832	1.00322
*A*24*	0.57155	0.39897	0.70518
*B*50.01*	0.57821	-0.39422	0.70904
*B*47.01*	0.59020	0.11571	0.21487
*DQB1*02.02*	0.59192	-0.05298	0.09883
*DRB1*13.03*	0.59369	0.13881	0.26019
*DPB1*20.01*	0.59399	0.13056	0.24492
*DPB1*11.01*	0.59437	0.21873	0.41076
*B*18*	0.59677	-0.53061	1.00296
*DPB1*16.01*	0.60248	0.13067	0.25089
*DRB1*14.01*	0.60752	0.18247	0.35526
*DQA1*03*	0.61245	0.13641	0.26928
*B*27.05*	0.63588	0.03316	0.07003

*B*07.02*	0.63769	-0.02654	0.05636
*DRB1*11.01*	0.64015	0.04651	0.09949
*C*07.01*	0.64988	-0.02642	0.05821
*B*44.03*	0.65822	0.08929	0.20183
*DQA1*03.03*	0.66055	0.05143	0.11711
*DRB1*14*	0.66672	0.43386	1.00745
*C*12.02*	0.67746	-0.41234	0.99136
*DRB1*04.03*	0.67917	-0.41511	1.00364
*DPB1*04.01*	0.68980	0.01555	0.03895
*DRB4*01.01*	0.69167	0.08326	0.20993
*A*29.02*	0.69278	-0.14992	0.37947
*C*07.04*	0.69738	0.05319	0.13679
*C*15.02*	0.70397	-0.04623	0.12167
*DRB1*14.54*	0.70767	0.07413	0.19769
*B*56.01*	0.70949	0.04973	0.13349
*A*02.01*	0.73725	0.01357	0.04044
*DQA1*06.01*	0.74108	-0.23479	0.71057
*DRB3*02.02*	0.74269	-0.02074	0.06318
*A*11.01*	0.74393	-0.03157	0.09663
*A*30.01*	0.74733	0.11486	0.35653
*B*27.02*	0.74741	0.16224	0.50375
*DRB1*16.01*	0.75431	-0.06883	0.21994
*B*58.01*	0.76677	0.09451	0.31863
*C*16.01*	0.77032	0.08851	0.30316
*A*32.01*	0.77706	-0.02870	0.10137
*B*44*	0.78317	0.19657	0.71433
*B*52.01*	0.78915	-0.26528	0.99204
*B*49.01*	0.79269	-0.15276	0.58124
*B*18.01*	0.80953	0.02094	0.08689

*DPB1*13.01*	0.81813	-0.08183	0.35583
*DPB1*02.01*	0.83998	-0.01119	0.05540
*B*44.27*	0.84467	0.04423	0.22576
*A*25.01*	0.85132	0.03258	0.17380
*B*41.01*	0.86148	-0.07168	0.41077
*B*57.01*	0.86521	0.02862	0.16860
*DPB1*05.01*	0.86986	-0.02099	0.12814
*DQB1*03.01*	0.87042	-0.01002	0.06141
*B*39.01*	0.87409	0.01611	0.10163
*C*07.02*	0.87558	-0.00847	0.05410
*C*03.02*	0.88813	0.04482	0.31864
*A*68.01*	0.89034	0.01099	0.07968
*C*17.01*	0.89067	-0.05647	0.41079
*C*12.03*	0.89400	-0.01448	0.10870
*B*35.02*	0.89758	-0.09075	0.70505
*A*33.01*	0.90612	-11.02764	93.50236
*DPB1*02.02*	0.90773	-11.04116	95.26317
*DQB1*02*	0.90963	-10.24297	90.23997
*A*03.01*	0.91671	0.00462	0.04423
*DRB1*15.02*	0.91674	-9.94433	95.12547
*DPB1*04.02*	0.91743	-0.00503	0.04855
*DQB1*06.01*	0.91964	-9.95221	98.64800
*A*02.02*	0.92292	-8.98224	92.83593
*C*15.05*	0.92293	-10.08148	104.20739
*A*02*	0.92303	-10.12313	104.76844
*B*15*	0.92533	-10.02786	106.99311
*B*13*	0.93090	-7.62758	87.96480
*DRB1*08.03*	0.93288	0.04896	0.58129
*B*45.01*	0.93303	0.04882	0.58094

*DPB1*06.01*	0.93340	-11.06228	132.37913
*B*39*	0.93375	-11.03916	132.79223
*A*29.01*	0.93500	-0.03096	0.37961
*C*12*	0.93554	-7.96440	98.47907
*DQB1*03.04*	0.93576	-9.02649	111.98702
*DRB1*01.03*	0.93779	-11.15696	142.95358
*DQB1*04*	0.93827	-7.04702	90.99216
*A*01*	0.93850	-7.15135	92.68700
*DQB1*05.02*	0.94115	0.01437	0.19459
*C*16.02*	0.94229	-6.82685	94.30449
*C*04*	0.94282	-9.21624	128.50294
*A*02.17*	0.94302	-7.44233	104.12351
*DRB1*15*	0.94574	-6.72151	98.75371
*B*07*	0.94634	0.04764	0.70786
*A*31*	0.94686	-8.21041	123.17890
*DPB1*09.01*	0.94839	-6.78905	104.87935
*A*28*	0.94877	-6.13535	95.48948
*A*11*	0.95023	-7.79247	124.85030
*B*41.02*	0.95025	-0.02559	0.41018
*A*30.02*	0.95194	-6.83480	113.40284
*A*02.06*	0.95250	-6.48119	108.81524
*A*01.02*	0.95342	-6.93748	118.75725
*A*68*	0.95343	-6.48296	111.00234
*B*15.16*	0.95421	-7.85175	136.72587
*B*40*	0.95438	-6.36736	111.30807
*DRB1*13*	0.95445	-6.30212	110.33891
*DQB1*06*	0.95578	-7.84965	141.56302
*C*01*	0.95731	-6.88303	128.57991
*B*08*	0.95733	-5.13542	95.97130

*B*27*	0.95796	-5.74072	108.89646
*C*03*	0.96115	-6.94272	142.53837
*A*32*	0.96176	-6.78794	141.56575
*A*68167*	0.96223	-5.16416	109.04544
*C*02*	0.96309	-6.33195	136.82868
*C*06*	0.96432	-6.14753	137.43337
*B*35.03*	0.96530	-0.03094	0.71119
*DPB1*34.01*	0.96599	-5.09320	119.46809
*C*05*	0.96638	-5.03582	119.46814
*DRB1*04.02*	0.97088	-4.92258	134.85461
*A*31.01*	0.97518	-0.00300	0.09658
*B*15.17*	0.97797	-0.01964	0.71133
*C*17.03*	0.98105	-0.00974	0.41018
*DRB5*02.02*	0.98274	0.00476	0.22005
*B*51.01*	0.99762	0.00024	0.08021

Large Registry Based Analysis of Genetic Predisposition to Tuberculosis
Identifies Genetic Risk Factors at HLA
Supplements

Table S7 – Epidemiological correlates

Trait	TB N	TB %	CTRL N	CTRL %	FG R7 N	FG R7 %	P unadj.	P adj.	beta (adj.)	OR (adj.)	SE adj.	CI 95% low	CI 95% high
Ever smoker	928	66.43%	95404	49.63%	96332	49.75%	1.30E-34	2.00E-16	0.63	1.87	0.06	1.75	1.99
Current smoker	598	42.81%	49898	25.96%	50496	26.08%	2.60E-44	2.00E-16	0.66	1.94	0.06	1.82	2.06
COPD	402	21.21%	12853	4.18%	13255	4.29%	1.40E-233	2.00E-16	1.31	3.71	0.07	3.57	3.85
Diabetes	377	19.89%	53011	17.25%	53388	17.27%	2.50E-03	9.40E-02	-0.11	0.89	0.07	0.75	1.03
Hypertension	324	17.10%	84667	27.56%	85491	27.65%	8.20E-52	5.60E-02	0.11	1.11	0.06	0.99	1.23
Asthma	377	19.89%	31974	10.41%	32351	10.46%	2.80E-163	2.00E-16	2.40	10.97	0.11	10.75	11.19
Major cardiac event (CHD)	784	41.37%	80123	26.08%	80907	26.17%	1.30E-49	3.80E-03	0.16	1.17	0.05	1.07	1.27
HIV	9	0.47%	418	0.14%	427	0.14%	2.10E-04	1.30E-01	0.87	2.40	0.58	1.26	3.54
Rheumatoid arthritis	122	6.44%	9733	3.17%	9855	3.19%	1.20E-18	1.10E-05	0.50	1.65	0.11	1.43	1.87
Biological medication for rheumatoid arthritis	23	1.21%	3116	1.01%	3139	1.02%	3.90E-01	1.10E-02	0.65	1.91	0.25	1.42	2.40
Sleep apnea	175	9.23%	27032	8.80%	27207	8.80%	5.10E-01	6.90E-01	0.04	1.10	0.09	0.92	1.28
Alcohol dependence	123	6.49%	8014	2.61%	8137	2.63%	5.20E-25	2.00E-16	0.91	2.50	0.10	2.30	2.70
Alcohol use disorder	157	8.28%	12505	4.07%	12662	4.10%	2.00E-19	1.10E-15	0.75	2.11	0.09	1.93	2.29
Inflammatory bowel disease	74	3.91%	8630	2.81%	8704	2.82%	4.20E-03	4.50E-05	0.53	1.71	0.13	1.46	1.96
Crohn's disease	12	0.63%	1533	0.50%	1545	0.50%	3.70E-01	2.00E-02	0.71	2.04	0.31	1.43	2.65
**Tuberculosis all organs**					**1895**	**0.61%**							

Trait = Studied risk factor or comorbidity
TB = Tuberculosis cases (ICD-10 A15-A19, all organs)
CTRL = Controls
FG = FinnGen data freeze R7
N = Number of individuals
% = Percentage of individuals with the studied trait (within TB cases, controls or FinnGen data freeze R7)

Adj. = Value comes from the adjusted multivariate logistic regression model (adjusted with age at death or end of follow up (12/31/2019), BMI, sex and the first 10 genetic principal components)
P = P-Value
Beta = Effect estimate
OR = Odds ratio
SE = Standard error
CI 95% = 95 % confidence intervals for odds ratio

Table S8 – Cox proportional hazards model

Trait	P-value unadj.	P-value adj.	SE	Coef.	HR	CI 95% low	CI 95% high	Global P-value of trait	N cases	N controls
Current smoker	2.00E-16	3.27E-10	0.176	0.900	2.461	1.742	3.476	0.039	596	798
Former smoker	2.61E-02	3.31E-01	0.168	0.163	1.177	0.847	1.635	0.039	330	1064
COPD	2.00E-16	8.98E-10	0.090	0.548	1.730	1.452	2.062	0.002	402	992
Asthma	9.76E-01	3.47E-01	0.108	0.101	1.107	0.896	1.367	0.037	377	1017
Major cardiac event (CHD)	2.90E-01	8.55E-01	0.093	-0.017	0.983	0.819	1.180	0.046	784	610
Rheumatoid arthritis	2.64E-01	4.43E-01	0.223	-0.171	0.843	0.544	1.310	0.068	78	1316
Alcohol dependence	2.67E-09	4.20E-07	0.160	0.807	2.240	1.640	3.060	0.090	100	1294
Alcohol use disorder	9.48E-09	1.45E-06	0.147	0.708	2.030	1.520	2.710	0.091	122	1272
Inflammatory bowel disease	8.22E-01	5.83E-01	0.271	-0.149	0.862	0.506	1.470	0.048	48	1346

Trait = Studied risk factor or comorbidity
N = Number of individuals
Adj. = Value comes from the adjusted Cox proportional hazards model (adjusted with stratified sex, stratified cohort (cohort representing for example biobank or study included within the FinnGen study), BMI and the first 10 genetic principal components)
P = P-Value
Coef. = Regression coefficient
HR = Hazard ratio
SE = Standard error
CI 95% = 95 % confidence intervals for hazard ratio

Table S9 – Changes of smoking over the decades

Description of smoking status among tuberculosis cases and matched controls throughout different decades starting from 1970s (time of the first diagnoses of tuberculosis in FinnGen data freeze R7).

**Decade**	**TB ever smoker (%)**	**Matched CTRL ever smoker (%)**
1970 - 1979	64.30 %	57.00 %
1980 - 1989	66.70 %	62.70 %
1990 - 1999	74.40 %	60.20 %
2000 - 2009	63.40 %	55.90 %
2010 - 2019	67.40 %	52.20 %
**Average**	**67.24 %**	**57.60 %**

TB = Tuberculosis (A15-A19, all organs)
CTRL = Control
Ever smoker = Smoking status "current" or "former"
% = Proportion of individuals who were ever smokers among cases or controls

*NB! Controls were matched according to age and sex with the tuberculosis cases using R programming language.

Table S10 – Genetic Correlation TB vs. AUD

Genetic correlation results for tuberculosis (TB) and alcohol use disorder (AUD). Genetic correlation was conducted using three different AUD traits: Alcohol Use Disorders Identification Test – Consumption (AUDIT-C), Alcohol Use Disorders Identification Test – Problematic consequences of drinking (AUDIT-P) and Alcohol Use Disorders Identification Test – Total score (AUDIT-T).

Trait 1	Trait 2	h2	Genetic Correlation	Z-score	P-value
TB	AUDIT-C	0.0839 (0.0055)	0.1 (0.1192)	0.8387	0.4016
TB	AUDIT-P	0.0584 (0.0048)	0.1378 (0.1431)	0.963	0.3355
TB	AUDIT-T	0.0919 (0.0059)	0.1024 (0.1176)	0.8708	0.3839

h2 = heritability of the trait 2

Large Registry Based Analysis of Genetic Predisposition to Tuberculosis Identifies Genetic Risk Factors at HLA
Supplements

Supplemental Information

Previous HLA findings from the literature

Our results align with previous studies showing the association between HLA class II region and tuberculosis:

An Icelandic GWAS study using cohort from Iceland, Croatia and Russia, showed susceptible association between tuberculosis and *HLA DQA1*03:01* allele [1]. In addition, different HLA class II alleles have been associated with tuberculosis in, for example, different Asian and African populations [2]. GWAS and HLA fine mapping in the Han Chinese population identified *HLA DQB1*02:01* having a significant association with tuberculosis [3]. Additionally, HLA class II alleles *HLA DRB1*04:01* (South African [4]), *HLA DRB1*15:01* (South African [4]), *HLA DRB1*11:01* (South African [4]), *HLA DRB1*09:01* (Southeast Asian [5]), *HLA DQB1*03:03* (Southeast Asian [5]), *HLA DRB1*01:01* (Korean [6] and Iranian [7]), *HLA DQA1*01:01* (Iranian [7] and Mexican [8]), *HLA DQB1*05:03* (Cambodian [9]), *HLA DQA1*05:05* (Chinese [10]) and gene *HLA DRA* (Indian [11]) have been associated with tuberculosis. In addition, *HLA DQA1*03* was associated with susceptibility of young onset tuberculosis in a Chinese cohort [12]. Our finding of association between tuberculosis and *HLA DQB1*05:01* have previously been shown in South Indian [13] and Mexican [8] cohorts, but not in Caucasians nor in an as large cohort as FinnGen is. Furthermore, Tian et al. (2017) showed HLA class II genome-wide association among individuals with self-reported positivity in tuberculosis skin test using the 23andMe-study and approximately 100 000 individuals of European ancestry [14].

References

1. Sveinbjornsson,G., Gudbjartsson,D.F., Halldorsson,B.V., Kristinsson,K.G., Gottfredsson,M., Barrett,J.C., Gudmundsson,I.J., Blondal,K., Gylfason,A., Gudjonsson,S.A., et al. (2016) HLA class II sequence variants influence tuberculosis risk in populations of European ancestry. *Nat. Genet.*, **48**, 318–322.
2. Oliveira-Cortez,A., Melo,A.C., Chaves,V.E., Condino-Neto,A. and Camargos,P. (2016) Do HLA class II genes protect against pulmonary tuberculosis? A systematic review and meta-analysis. *Eur. J. Clin. Microbiol. Infect. Dis.*, **35**, 1567–1580.
3. Qi,H., Zhang,Y.B., Sun,L., Chen,C., Xu,B., Xu,F., Liu,J.W., Liu,J.C., Chen,C., Jiao,W.W., et al. (2017) Discovery of susceptibility loci associated with tuberculosis in Han Chinese. *Hum. Mol. Genet.*, **26**, 4752–4763.

4. Seedat,F., James,I., Loubser,S., Waja,Z., Mallal,S.A., Hoffmann,C., Tiemessen,C.T., Chaisson,R.E. and Martinson,N.A. (2021) Human leukocyte antigen associations with protection against tuberculosis infection and disease in human immunodeficiency virus-1 infected individuals, despite household tuberculosis exposure and immune suppression. *Tuberculosis (Edinb)*, **126**, 102023.
5. Toyo-Oka,L., Mahasirimongkol,S., Yanai,H., Mushiroda,T., Wattanapokayakit,S., Wichukchinda,N., Yamada,N., Smittipat,N., Juthayothin,T., Palittapongarnpim,P., et al. (2017) Strain-based HLA association analysis identified HLA-DRB1*09:01 associated with modern strain tuberculosis. *HLA*, **90**, 149–156.
6. Kim,H.S., Park,M.H., Song,E.Y., Park,H., Kwon,S.Y., Han,S.K. and Shim,Y.S. (2005) Association of HLA-DR and HLA-DQ genes with susceptibility to pulmonary tuberculosis in Koreans: preliminary evidence of associations with drug resistance, disease severity, and disease recurrence. *Hum. Immunol.*, **66**, 1074–1081.
7. Amirzargar,A.A., Yalda,A., Hajabolbaghi,M., Khosravi,F., Jabbari,H., Rezaei,N., Niknam,M.H., Ansari,B., Moradi,B. and Nikbin,B. (2004) The association of HLA-DRB, DQA1, DQB1 alleles and haplotype frequency in Iranian patients with pulmonary tuberculosis. *Int. J. Tuberc. Lung. Dis.*, **8**, 1017–1021.
8. Terán-Escandón,D., Terán-Ortiz,L., Camarena-Olvera,A., González-Avila,G., Vaca-Marín,M.A., Granados,J. and Selma,M. (1999) Human leukocyte antigen-associated susceptibility to pulmonary tuberculosis: molecular analysis of class II alleles by DNA amplification and oligonucleotide hybridization in Mexican patients. *Chest*, **115**, 428–433.
9. Goldfeld,A.E., Delgado,J.C., Thim,S., Bozon,M.V., Uglialoro,A.M., Turbay,D., Cohen,C. and Yunis,E.J. (1998) Association of an HLA-DQ allele with clinical tuberculosis. *JAMA*, **279**, 226–228.
10. Li, M., Hu, Y., Zhao, B., Chen, L., Huang, H., Huai, C., Zhang, X., Zhang, J., Zhou, W., Shen, L., et al. (2021) A next generation sequencing combined genome-wide association study identifies novel tuberculosis susceptibility loci in Chinese population. *Genomics*, **113**, 2377–2384.
11. Bhattacharyya,C., Majumder,P.P. and Pandit,B. (2019) An exome wide association study of pulmonary tuberculosis patients and their asymptomatic household contacts. *Infect. Genet. Evol.*, **71**, 76–81.
12. Tang,N.L., Wang,X., Chang,K.C., Chan,C.Y., Szeto,N.W., Huang,D., Wu,J., Lui,G.C.Y., Leung,C.C. and Hui,M. (2019) Genetic susceptibility to Tuberculosis: Interaction between HLA-DQA1 and age of onset. *Infect. Genet. Evol.*, **68**, 98–104.
13. Ravikumar,M., Dheenadhayalan,V., Rajaram,K., Lakshmi,S.S., Kumaran,P.P., Paramasivan,C.N., Balakrishnan,K. and Pitchappan,R.M. (1999) Associations of HLA-DRB1, DQB1 and DPB1 alleles with pulmonary tuberculosis in south India. *Tuber. Lung. Dis.*, **79**, 309–317.
14. Tian,C., Hromatka,B.S., Kiefer,A.K., Eriksson,N., Noble,S.M., Tung,J.Y. and Hind,D.A. (2017) Genome-wide association and HLA region fine-mapping studies identify susceptibility loci for multiple common infections. *Nat. Commun.*, **8**, 599.

Large Registry Based Analysis of Genetic Predisposition to Tuberculosis
Identifies Genetic Risk Factors at HLA
Supplements

Contributors of FinnGen

Steering Committee

Aarno Palotie	Institute for Molecular Medicine Finland, HiLIFE, University of Helsinki, Finland
Mark Daly	Institute for Molecular Medicine Finland, HiLIFE, University of Helsinki, Finland

Pharmaceutical companies

Bridget Riley-Gills	Abbvie, Chicago, IL, United States
Howard Jacob	Abbvie, Chicago, IL, United States
Dirk Paul	Astra Zeneca, Cambridge, United Kingdom
Heiko Runz	Biogen, Cambridge, MA, United States
Sally John	Biogen, Cambridge, MA, United States
George Okafo	Boehringer Ingelheim, Ingelheim am Rhein, Germany
Nathan Lawless	Boehringer Ingelheim, Ingelheim am Rhein, Germany
Robert Plenge	Celgene, Summit, NJ, United States/Bristol Myers Squibb, New York, NY, United States
Joseph Maranville	Celgene, Summit, NJ, United States/Bristol Myers Squibb, New York, NY, United States
Mark McCarthy	Genentech, San Francisco, CA, United States
Julie Hunkapiller	Genentech, San Francisco, CA, United States
Meg Ehm	GlaxoSmithKline, Brentford, United Kingdom
Kirsi Auro	GlaxoSmithKline, Brentford, United Kingdom
Simonne Longerich	Merck, Kenilworth, NJ, United States
Caroline Fox	Merck, Kenilworth, NJ, United States
Anders Mälarstig	Pfizer, New York, NY, United States
Katherine Klinger	Sanofi, Paris, France
Deepak Raipal	Sanofi, Paris, France
Eric Green	Maze Therapeutics, San Francisco, CA, United States

Large Registry Based Analysis of Genetic Predisposition to Tuberculosis
Identifies Genetic Risk Factors at HLA
Supplements

Robert Graham	Maze Therapeutics, San Francisco, CA, United States
Robert Yang	Janssen Biotech, Beerse, Belgium
Chris O´Donnell	Novartis, Basel, Switzerland

University of Helsinki & Biobanks

Tomi Mäkelä	HiLIFE, University of Helsinki, Finland, Finland
Jaakko Kaprio	Institute for Molecular Medicine Finland, HiLIFE, Helsinki, Finland, Finland
Petri Virolainen	Auria Biobank / University of Turku / Hospital District of Southwest Finland, Turku, Finland
Antti Hakanen	Auria Biobank / University of Turku / Hospital District of Southwest Finland, Turku, Finland
Terhi Kilpi	THL Biobank / The National Institute of Health and Welfare Helsinki, Finland
Markus Perola	THL Biobank / The National Institute of Health and Welfare Helsinki, Finland
Jukka Partanen	Finnish Red Cross Blood Service / Finnish Hematology Registry and Clinical Biobank, Helsinki, Finland
Anne Pitkäranta	Helsinki Biobank / Helsinki University and Hospital District of Helsinki and Uusimaa, Helsinki
Juhani Junttila	Northern Finland Biobank Borealis / University of Oulu / Northern Ostrobothnia Hospital District, Oulu, Finland
Raisa Serpi	Northern Finland Biobank Borealis / University of Oulu / Northern Ostrobothnia Hospital District, Oulu, Finland
Tarja Laitinen	Finnish Clinical Biobank Tampere / University of Tampere / Pirkanmaa Hospital District, Tampere, Finland
Veli-Matti Kosma	Biobank of Eastern Finland / University of Eastern Finland / Northern Savo Hospital District, Kuopio, Finland
Jari Laukkanen	Central Finland Biobank / University of Jyväskylä / Central Finland Health Care District, Jyväskylä, Finland
Marco Hautalahti	FINBB - Finnish biobank cooperative

Other Experts/ Non-Voting Members

Outi Tuovila	Business Finland, Helsinki, Finland

Large Registry Based Analysis of Genetic Predisposition to Tuberculosis Identifies Genetic Risk Factors at HLA
Supplements

Raimo Pakkanen Business Finland, Helsinki, Finland

Large Registry Based Analysis of Genetic Predisposition to Tuberculosis Identifies Genetic Risk Factors at HLA
Supplements

Scientific Committee

Pharmaceutical companies

Jeffrey Waring	Abbvie, Chicago, IL, United States
Bridget Riley-Gillis	Abbvie, Chicago, IL, United States
Ioanna Tachmazidou	Astra Zeneca, Cambridge, United Kingdom
Chia-Yen Chen	Biogen, Cambridge, MA, United States
Heiko Runz	Biogen, Cambridge, MA, United States
Zhihao Ding	Boehringer Ingelheim, Ingelheim am Rhein, Germany
Marc Jung	Boehringer Ingelheim, Ingelheim am Rhein, Germany
Shameek Biswas	Celgene, Summit, NJ, United States/Bristol Myers Squibb, New York, NY, United States
Sarah Pendergrass	Genentech, San Francisco, CA, United States
Julie Hunkapiller	Genentech, San Francisco, CA, United States
Meg Ehm	GlaxoSmithKline, Brentford, United Kingdom
David Pulford	GlaxoSmithKline, Brentford, United Kingdom
Neha Raghavan	Merck, Kenilworth, NJ, United States
Adriana Huertas-Vazquez	Merck, Kenilworth, NJ, United States
Jae-Hoon Sul	Merck, Kenilworth, NJ, United States
Anders Mälarstig	Pfizer, New York, NY, United States
Xinli Hu	Pfizer, New York, NY, United States
Katherine Klinger	Sanofi, Paris, France
Matthias Gossel	Sanofi, Paris, France
Robert Graham	Maze Therapeutics, San Francisco, CA, United States
Eric Green	Maze Therapeutics, San Francisco, CA, United States
Sahar Mozaffari	Maze Therapeutics, San Francisco, CA, United States
Dawn Waterworth	Janssen Research & Development, LLC, Spring House, PA, United States
Nicole Renaud	Novartis, Basel, Switzerland
Ma´en Obeidat	Novartis, Basel, Switzerland

Large Registry Based Analysis of Genetic Predisposition to Tuberculosis
Identifies Genetic Risk Factors at HLA
Supplements

University of Helsinki & Biobanks

Samuli Ripatti	Institute for Molecular Medicine Finland, HiLIFE, Helsinki, Finland
Johanna Schleutker	Auria Biobank / Univ. of Turku / Hospital District of Southwest Finland, Turku, Finland
Markus Perola	THL Biobank / The National Institute of Health and Welfare Helsinki, Finland
Mikko Arvas	Finnish Red Cross Blood Service / Finnish Hematology Registry and Clinical Biobank, Helsinki, Finland
Olli Carpén	Helsinki Biobank / Helsinki University and Hospital District of Helsinki and Uusimaa, Helsinki
Reetta Hinttala	Northern Finland Biobank Borealis / University of Oulu / Northern Ostrobothnia Hospital District, Oulu, Finland
Johannes Kettunen	Northern Finland Biobank Borealis / University of Oulu / Northern Ostrobothnia Hospital District, Oulu, Finland
Arto Mannermaa	Biobank of Eastern Finland / University of Eastern Finland / Northern Savo Hospital District, Kuopio, Finland
Katriina Aalto-Setälä	Finnish Clinical Biobank Tampere / University of Tampere / Pirkanmaa Hospital District, Tampere, Finland
Mika Kähönen	Finnish Clinical Biobank Tampere / University of Tampere / Pirkanmaa Hospital District, Tampere, Finland
Jari Laukkanen	Central Finland Biobank / University of Jyväskylä / Central Finland Health Care District, Jyväskylä, Finland
Johanna Mäkelä	FINBB - Finnish biobank cooperative

Clinical Groups

<u>Neurology Group</u>

Reetta Kälviäinen	Northern Savo Hospital District, Kuopio, Finland
Valtteri Julkunen	Northern Savo Hospital District, Kuopio, Finland
Hilkka Soininen	Northern Savo Hospital District, Kuopio, Finland
Anne Remes	Northern Ostrobothnia Hospital District, Oulu, Finland
Mikko Hiltunen	Northern Savo Hospital District, Kuopio, Finland
Jukka Peltola	Pirkanmaa Hospital District, Tampere, Finland

Large Registry Based Analysis of Genetic Predisposition to Tuberculosis Identifies Genetic Risk Factors at HLA
Supplements

Minna Raivio	Hospital District of Helsinki and Uusimaa, Helsinki, Finland
Pentti Tienari	Hospital District of Helsinki and Uusimaa, Helsinki, Finland
Juha Rinne	Hospital District of Southwest Finland, Turku, Finland
Roosa Kallionpää	Hospital District of Southwest Finland, Turku, Finland
Juulia Partanen	Institute for Molecular Medicine Finland, HiLIFE, University of Helsinki, Finland
Ali Abbasi	Abbvie, Chicago, IL, United States
Adam Ziemann	Abbvie, Chicago, IL, United States
Jeffrey Waring	Abbvie, Chicago, IL, United States
Nizar Smaoui	Abbvie, Chicago, IL, United States
Anne Lehtonen	Abbvie, Chicago, IL, United States
Susan Eaton	Biogen, Cambridge, MA, United States
Heiko Runz	Biogen, Cambridge, MA, United States
Sanni Lahdenperä	Biogen, Cambridge, MA, United States
Janet van Adelsberg	Celgene, Summit, NJ, United States/ Bristol Myers Squibb, New York, NY, United States
Shameek Biswas	Celgene, Summit, NJ, United States/ Bristol Myers Squibb, New York, NY, United States
Julie Hunkapiller	Genentech, San Francisco, CA, United States
Natalie Bowers	Genentech, San Francisco, CA, United States
Edmond Teng	Genentech, San Francisco, CA, United States
Sarah Pendergrass	Genentech, San Francisco, CA, United States
Fanli Xu	GlaxoSmithKline, Brentford, United Kingdom
David Pulford	GlaxoSmithKline, Brentford, United Kingdom
Kirsi Auro	GlaxoSmithKline, Brentford, United Kingdom
Laura Addis	GlaxoSmithKline, Brentford, United Kingdom
John Eicher	GlaxoSmithKline, Brentford, United Kingdom
Qingqin S Li	Janssen Research & Development, LLC, Titusville, NJ 08560, United States
Karen He	Janssen Research & Development, LLC, Spring House, PA, United States

Ekaterina Khramtsova	Janssen Research & Development, LLC, Spring House, PA, United States
Beryl Cummings	Maze Therapeutics, San Francisco, CA, United States
Neha Raghavan	Merck, Kenilworth, NJ, United States
Kari Linden	Pfizer, New York, NY, United States

<u>Gastroenterology Group</u>

Martti Färkkilä	Hospital District of Helsinki and Uusimaa, Helsinki, Finland
Jukka Koskela	Hospital District of Helsinki and Uusimaa, Helsinki, Finland
Sampsa Pikkarainen	Hospital District of Helsinki and Uusimaa, Helsinki, Finland
Airi Jussila	Pirkanmaa Hospital District, Tampere, Finland
Katri Kaukinen	Pirkanmaa Hospital District, Tampere, Finland
Timo Blomster	Northern Ostrobothnia Hospital District, Oulu, Finland
Mikko Kiviniemi	Northern Savo Hospital District, Kuopio, Finland
Markku Voutilainen	Hospital District of Southwest Finland, Turku, Finland
Mark Daly	Institute for Molecular Medicine Finland, HiLIFE, University of Helsinki, Finland
Ali Abbasi	Abbvie, Chicago, IL, United States
Graham Heap	Abbvie, Chicago, IL, United States
Jeffrey Waring	Abbvie, Chicago, IL, United States
Nizar Smaoui	Abbvie, Chicago, IL, United States
Fedik Rahimov	Abbvie, Chicago, IL, United States
Anne Lehtonen	Abbvie, Chicago, IL, United States
Keith Usiskin	Celgene, Summit, NJ, United States/ Bristol Myers Squibb, New York, NY, United States
Tim Lu	Genentech, San Francisco, CA, United States
Natalie Bowers	Genentech, San Francisco, CA, United States
Danny Oh	Genentech, San Francisco, CA, United States
Sarah Pendergrass	Genentech, San Francisco, CA, United States
Linda McCarthy	GlaxoSmithKline, Brentford, United Kingdom

Large Registry Based Analysis of Genetic Predisposition to Tuberculosis
Identifies Genetic Risk Factors at HLA
Supplements

Amy Hart	Janssen Research & Development, LLC, Spring House, PA, United States
Meijian Guan	Janssen Research & Development, LLC, Spring House, PA, United States
Jason Miller	Merck, Kenilworth, NJ, United States
Kirsi Kalpala	Pfizer, New York, NY, United States
Melissa Miller	Pfizer, New York, NY, United States
Xinli Hu	Pfizer, New York, NY, United States

Rheumatology Group

Kari Eklund	Hospital District of Helsinki and Uusimaa, Helsinki, Finland
Antti Palomäki	Hospital District of Southwest Finland, Turku, Finland
Pia Isomäki	Pirkanmaa Hospital District, Tampere, Finland
Laura Pirilä	Hospital District of Southwest Finland, Turku, Finland
Oili Kaipiainen-Seppänen	Northern Savo Hospital District, Kuopio, Finland
Johanna Huhtakangas	Northern Ostrobothnia Hospital District, Oulu, Finland
Nina Mars	Institute for Molecular Medicine Finland, HiLIFE, Helsinki, Finland
Ali Abbasi	Abbvie, Chicago, IL, United States
Jeffrey Waring	Abbvie, Chicago, IL, United States
Fedik Rahimov	Abbvie, Chicago, IL, United States
Apinya Lertratanakul	Abbvie, Chicago, IL, United States
Nizar Smaoui	Abbvie, Chicago, IL, United States
Anne Lehtonen	Abbvie, Chicago, IL, United States
David Close	Astra Zeneca, Cambridge, United Kingdom
Marla Hochfeld	Celgene, Summit, NJ, United States/ Bristol Myers Squibb, New York, NY, United States
Natalie Bowers	Genentech, San Francisco, CA, United States
Sarah Pendergrass	Genentech, San Francisco, CA, United States
Jorge Esparza Gordillo	GlaxoSmithKline, Brentford, United Kingdom
Kirsi Auro	GlaxoSmithKline, Brentford, United Kingdom

Dawn Waterworth	Janssen Research & Development, LLC, Spring House, PA, United States
Fabiana Farias	Merck, Kenilworth, NJ, United States
Kirsi Kalpala	Pfizer, New York, NY, United States
Nan Bing	Pfizer, New York, NY, United States
Xinli Hu	Pfizer, New York, NY, United States

Pulmonology Group

Tarja Laitinen	Pirkanmaa Hospital District, Tampere, Finland
Margit Pelkonen	Northern Savo Hospital District, Kuopio, Finland
Paula Kauppi	Hospital District of Helsinki and Uusimaa, Helsinki, Finland
Hannu Kankaanranta	University of Gothenburg, Gothenburg, Sweden/ Seinäjoki Central Hospital, Seinäjoki, Finland/ Tampere University, Tampere, Finland
Terttu Harju	Northern Ostrobothnia Hospital District, Oulu, Finland
Riitta Lahesmaa	Hospital District of Southwest Finland, Turku, Finland

Nizar Smaoui	Abbvie, Chicago, IL, United States
Alex Mackay	Astra Zeneca, Cambridge, United Kingdom
Glenda Lassi	Astra Zeneca, Cambridge, United Kingdom
Susan Eaton	Biogen, Cambridge, MA, United States
Steven Greenberg	Celgene, Summit, NJ, United States/ Bristol Myers Squibb, New York, NY, United States
Hubert Chen	Genentech, San Francisco, CA, United States
Sarah Pendergrass	Genentech, San Francisco, CA, United States
Natalie Bowers	Genentech, San Francisco, CA, United States
Joanna Betts	GlaxoSmithKline, Brentford, United Kingdom
Soumitra Ghosh	GlaxoSmithKline, Brentford, United Kingdom
Kirsi Auro	GlaxoSmithKline, Brentford, United Kingdom
Rajashree Mishra	GlaxoSmithKline, Brentford, United Kingdom
Majd Mouded	Novartis, Basel, Switzerland
Debby Ngo	Novartis, Basel, Switzerland

Large Registry Based Analysis of Genetic Predisposition to Tuberculosis
Identifies Genetic Risk Factors at HLA
Supplements

<u>Cardiometabolic Diseases Group</u>

Teemu Niiranen	The National Institute of Health and Welfare Helsinki, Finland
Felix Vaura	The National Institute of Health and Welfare Helsinki, Finland
Veikko Salomaa	The National Institute of Health and Welfare Helsinki, Finland
Kaj Metsärinne	Hospital District of Southwest Finland, Turku, Finland
Jenni Aittokallio	Hospital District of Southwest Finland, Turku, Finland
Mika Kähönen	Pirkanmaa Hospital District, Tampere, Finland
Jussi Hernesniemi	Pirkanmaa Hospital District, Tampere, Finland
Juhani Junttila	Northern Ostrobothnia Hospital District, Oulu, Finland
Markku Laakso	Northern Savo Hospital District, Kuopio, Finland
Jussi Pihlajamäki	Northern Savo Hospital District, Kuopio, Finland
Daniel Gordin	Hospital District of Helsinki and Uusimaa, Helsinki, Finland
Juha Sinisalo	Hospital District of Helsinki and Uusimaa, Helsinki, Finland
Marja-Riitta Taskinen	Hospital District of Helsinki and Uusimaa, Helsinki, Finland
Tiinamaija Tuomi	Hospital District of Helsinki and Uusimaa, Helsinki, Finland
Timo Hiltunen	Hospital District of Helsinki and Uusimaa, Helsinki, Finland
Jari Laukkanen	Central Finland Health Care District, Jyväskylä, Finland
Amanda Elliott	Institute for Molecular Medicine Finland, HiLIFE, University of Helsinki, Finland / Broad Institute, Cambridge, MA, United States
Mary Pat Reeve	Institute for Molecular Medicine Finland, HiLIFE, University of Helsinki, Finland
Sanni Ruotsalainen	Institute for Molecular Medicine Finland, HiLIFE, University of Helsinki, Finland
Benjamin Challis	Astra Zeneca, Cambridge, United Kingdom
Dirk Paul	Astra Zeneca, Cambridge, United Kingdom
Keith Usiskin	Celgene, Summit, NJ, United States/ Bristol Myers Squibb, New York, NY, United States
Julie Hunkapiller	Genentech, San Francisco, CA, United States
Natalie Bowers	Genentech, San Francisco, CA, United States
Sarah Pendergrass	Genentech, San Francisco, CA, United States

Audrey Chu	GlaxoSmithKline, Brentford, United Kingdom
Kirsi Auro	GlaxoSmithKline, Brentford, United Kingdom
Dermot Reilly	Janssen Research & Development, LLC, Boston, MA, United States
Beryl Cummings	Maze Therapeutics, San Francisco, CA, United States
Mike Mendelson	Novartis, Boston, MA, United States
Jaakko Parkkinen	Pfizer, New York, NY, United States
Melissa Miller	Pfizer, New York, NY, United States

Oncology Group

Tuomo Meretoja	Hospital District of Helsinki and Uusimaa, Helsinki, Finland
Heikki Joensuu	Hospital District of Helsinki and Uusimaa, Helsinki, Finland
Olli Carpén	Hospital District of Helsinki and Uusimaa, Helsinki, Finland
Lauri Aaltonen	Hospital District of Helsinki and Uusimaa, Helsinki, Finland
Johanna Mattson	Hospital District of Helsinki and Uusimaa, Helsinki, Finland
Eveliina Salminen	Hospital District of Helsinki and Uusimaa, Helsinki, Finland
Annika Auranen	Pirkanmaa Hospital District , Tampere, Finland
Peeter Karihtala	Northern Ostrobothnia Hospital District, Oulu, Finland
Päivi Auvinen	Northern Savo Hospital District, Kuopio, Finland
Klaus Elenius	Hospital District of Southwest Finland, Turku, Finland
Johanna Schleutker	Hospital District of Southwest Finland, Turku, Finland
Esa Pitkänen	Institute for Molecular Medicine Finland, HiLIFE, University of Helsinki, Finland
Nina Mars	Institute for Molecular Medicine Finland, HiLIFE, University of Helsinki, Finland
Mark Daly	Institute for Molecular Medicine Finland, HiLIFE, University of Helsinki, Finland
Relja Popovic	Abbvie, Chicago, IL, United States
Jeffrey Waring	Abbvie, Chicago, IL, United States
Bridget Riley-Gillis	Abbvie, Chicago, IL, United States
Anne Lehtonen	Abbvie, Chicago, IL, United States
Jennifer Schutzman	Genentech, San Francisco, CA, United States

Large Registry Based Analysis of Genetic Predisposition to Tuberculosis
Identifies Genetic Risk Factors at HLA
Supplements

Julie Hunkapiller	Genentech, San Francisco, CA, United States
Natalie Bowers	Genentech, San Francisco, CA, United States
Sarah Pendergrass	Genentech, San Francisco, CA, United States
Diptee Kulkarni	GlaxoSmithKline, Brentford, United Kingdom
Kirsi Auro	GlaxoSmithKline, Brentford, United Kingdom
Alessandro Porello	Janssen Research & Development, LLC, Spring House, PA, United States
Andrey Loboda	Merck, Kenilworth, NJ, United States
Heli Lehtonen	Pfizer, New York, NY, United States
Stefan McDonough	Pfizer, New York, NY, United States
Marika Crohns	Sanofi, Paris, France
Sauli Vuoti	Sanofi, Paris, France

Opthalmology Group

Kai Kaarniranta	Northern Savo Hospital District, Kuopio, Finland
Joni A Turunen	Hospital District of Helsinki and Uusimaa, Helsinki, Finland
Terhi Ollila	Hospital District of Helsinki and Uusimaa, Helsinki, Finland
Hannu Uusitalo	Pirkanmaa Hospital District, Tampere, Finland
Juha Karjalainen	Institute for Molecular Medicine Finland, HiLIFE, University of Helsinki, Finland
Esa Pitkänen	Institute for Molecular Medicine Finland, HiLIFE, University of Helsinki, Finland

Mengzhen Liu	Abbvie, Chicago, IL, United States
Heiko Runz	Biogen, Cambridge, MA, United States
Stephanie Loomis	Biogen, Cambridge, MA, United States
Erich Strauss	Genentech, San Francisco, CA, United States
Natalie Bowers	Genentech, San Francisco, CA, United States
Hao Chen	Genentech, San Francisco, CA, United States
Sarah Pendergrass	Genentech, San Francisco, CA, United States

Large Registry Based Analysis of Genetic Predisposition to Tuberculosis
Identifies Genetic Risk Factors at HLA
Supplements

Dermatology Group

Kaisa Tasanen	Northern Ostrobothnia Hospital District, Oulu, Finland
Laura Huilaja	Northern Ostrobothnia Hospital District, Oulu, Finland
Katariina Hannula-Jouppi	Hospital District of Helsinki and Uusimaa, Helsinki, Finland
Teea Salmi	Pirkanmaa Hospital District, Tampere, Finland
Sirkku Peltonen	Hospital District of Southwest Finland, Turku, Finland
Leena Koulu	Hospital District of Southwest Finland, Turku, Finland
Nizar Smaoui	Abbvie, Chicago, IL, United States
Fedik Rahimov	Abbvie, Chicago, IL, United States
Anne Lehtonen	Abbvie, Chicago, IL, United States
David Choy	Genentech, San Francisco, CA, United States
Sarah Pendergrass	Genentech, San Francisco, CA, United States
Dawn Waterworth	Janssen Research & Development, LLC, Spring House, PA, United States
Kirsi Kalpala	Pfizer, New York, NY, United States
Ying Wu	Pfizer, New York, NY, United States

Odontology Group

Pirkko Pussinen	Hospital District of Helsinki and Uusimaa, Helsinki, Finland
Aino Salminen	Hospital District of Helsinki and Uusimaa, Helsinki, Finland
Tuula Salo	Hospital District of Helsinki and Uusimaa, Helsinki, Finland
David Rice	Hospital District of Helsinki and Uusimaa, Helsinki, Finland
Pekka Nieminen	Hospital District of Helsinki and Uusimaa, Helsinki, Finland
Ulla Palotie	Hospital District of Helsinki and Uusimaa, Helsinki, Finland
Maria Siponen	Northern Savo Hospital District, Kuopio, Finland
Liisa Suominen	Northern Savo Hospital District, Kuopio, Finland
Päivi Mäntylä	Northern Savo Hospital District, Kuopio, Finland
Ulvi Gursoy	Hospital District of Southwest Finland, Turku, Finland

Vuokko Anttonen	Northern Ostrobothnia Hospital District, Oulu, Finland
Kirsi Sipilä	Northern Ostrobothnia Hospital District, Oulu, Finland
Sarah Pendergrass	Genentech, San Francisco, CA, United States

Women's Health and Reproduction Group

Hannele Laivuori	Institute for Molecular Medicine Finland, HiLIFE, University of Helsinki, Finland
Venla Kurra	Pirkanmaa Hospital District, Tampere, Finland
Laura Kotaniemi-Talonen	Pirkanmaa Hospital District, Tampere, Finland
Oskari Heikinheimo	Hospital District of Helsinki and Uusimaa, Helsinki, Finland
Ilkka Kalliala	Hospital District of Helsinki and Uusimaa, Helsinki, Finland
Lauri Aaltonen	Hospital District of Helsinki and Uusimaa, Helsinki, Finland
Varpu Jokimaa	Hospital District of Southwest Finland, Turku, Finland
Johannes Kettunen	Northern Ostrobothnia Hospital District, Oulu, Finland
Marja Vääräsmäki	Northern Ostrobothnia Hospital District, Oulu, Finland
Outi Uimari	Northern Ostrobothnia Hospital District, Oulu, Finland
Laure Morin-Papunen	Northern Ostrobothnia Hospital District, Oulu, Finland
Maarit Niinimäki	Northern Ostrobothnia Hospital District, Oulu, Finland
Terhi Piltonen	Northern Ostrobothnia Hospital District, Oulu, Finland
Katja Kivinen	Institute for Molecular Medicine Finland, HiLIFE, University of Helsinki, Finland
Elisabeth Widen	Institute for Molecular Medicine Finland, HiLIFE, University of Helsinki, Finland
Taru Tukiainen	Institute for Molecular Medicine Finland, HiLIFE, University of Helsinki, Finland
Mary Pat Reeve	Institute for Molecular Medicine Finland, HiLIFE, University of Helsinki, Finland
Mark Daly	Institute for Molecular Medicine Finland, HiLIFE, University of Helsinki, Finland
Liu Aoxing	Institute for Molecular Medicine Finland, HiLIFE, University of Helsinki, Finland

Large Registry Based Analysis of Genetic Predisposition to Tuberculosis
Identifies Genetic Risk Factors at HLA
Supplements

Andrea Ganna	Institute for Molecular Medicine Finland, HiLIFE, University of Helsinki, Finland
Niko Välimäki	University of Helsinki, Helsinki, Finland
Eija Laakkonen	University of Jyväskylä, Jyväskylä, Finland
Jaakko Tyrmi	University of Oulu, Oulu, Finland / University of Tampere, Tampere, Finland
Heidi Silven	University of Oulu, Oulu, Finland
Eeva Slitz	University of Oulu, Oulu, Finland
Riikka Arffman	University of Oulu, Oulu, Finland
Susanna Savukoski	University of Oulu, Oulu, Finland
Triin Laisk	Estonian biobank, Tartu, Estonia
Natalia Pujol	Estonian biobank, Tartu, Estonia
Bridget Riley-Gillis	Abbvie, Chicago, IL, United States
Mengzhen Liu	Abbvie, Chicago, IL, United States
Sarah Pendergrass	Genentech, San Francisco, CA, United States
Janet Kumar	GlaxoSmithKline, Brentford, United Kingdom
Kirsi Auro	GlaxoSmithKline, Brentford, United Kingdom

FinnGen Analysis working group

Bridget Riley-Gillis	Abbvie, Chicago, IL, United States
Reza Hammond	Abbvie, Chicago, IL, United States
Fedik Rahimov	Abbvie, Chicago, IL, United States
Sabah Kadri	Abbvie, Chicago, IL, United States
Mengzhen Liu	Abbvie, Chicago, IL, United States
Slavé Petrovski	Astra Zeneca, Cambridge, United Kingdom
Eleonor Wigmore	Astra Zeneca, Cambridge, United Kingdom
Adele Mitchell	Biogen, Cambridge, MA, United States
Benjamin Sun	Biogen, Cambridge, MA, United States
Ellen Tsai	Biogen, Cambridge, MA, United States

Large Registry Based Analysis of Genetic Predisposition to Tuberculosis Identifies Genetic Risk Factors at HLA
Supplements

Denis Baird	Biogen, Cambridge, MA, United States
Paola Bronson	Biogen, Cambridge, MA, United States
Ruoyu Tian	Biogen, Cambridge, MA, United States
Stephanie Loomis	Biogen, Cambridge, MA, United States
Yunfeng Huang	Biogen, Cambridge, MA, United States
Till Andlauer	Boehringer Ingelheim, Ingelheim am Rhein, Germany
Jatin Arora	Boehringer Ingelheim, Ingelheim am Rhein, Germany
Ghadi Rai	Boehringer Ingelheim, Ingelheim am Rhein, Germany
Zhihao Ding	Boehringer Ingelheim, Ingelheim am Rhein, Germany
Lorenz Maier	Boehringer Ingelheim, Ingelheim am Rhein, Germany
Karsten Quast	Boehringer Ingelheim, Ingelheim am Rhein, Germany
Francisco Herruzo	Boehringer Ingelheim, Ingelheim am Rhein, Germany
Daniel Lopez	Boehringer Ingelheim, Ingelheim am Rhein, Germany
Marc Jung	Boehringer Ingelheim, Ingelheim am Rhein, Germany
Boris Bartholdy	Boehringer Ingelheim, Ingelheim am Rhein, Germany
Joseph Maranville	Celgene, Summit, NJ, United States/ Bristol Myers Squibb, New York, NY, United States
Shameek Biswas	Celgene, Summit, NJ, United States/ Bristol Myers Squibb, New York, NY, United States
Elmutaz Mohammed	Celgene, Summit, NJ, United States/ Bristol Myers Squibb, New York, NY, United States
Samir Wadhawan	Celgene, Summit, NJ, United States/ Bristol Myers Squibb, New York, NY, United States
Erika Kvikstad	Celgene, Summit, NJ, United States/ Bristol Myers Squibb, New York, NY, United States
Minal Caliskan	Celgene, Summit, NJ, United States/ Bristol Myers Squibb, New York, NY, United States
Diana Chang	Genentech, San Francisco, CA, United States
Julie Hunkapiller	Genentech, San Francisco, CA, United States
Tushar Bhangale	Genentech, San Francisco, CA, United States
Natalie Bowers	Genentech, San Francisco, CA, United States
Sarah Pendergrass	Genentech, San Francisco, CA, United States

Large Registry Based Analysis of Genetic Predisposition to Tuberculosis Identifies Genetic Risk Factors at HLA
Supplements

Karen S King	GlaxoSmithKline, Brentford, United Kingdom
Padhraig Gormley	GlaxoSmithKline, Brentford, United Kingdom
Jimmy Liu	GlaxoSmithKline, Brentford, United Kingdom
Karsten Sieber	Janssen Research & Development, LLC, Spring House, PA, United States
Amy Hart	Janssen Research & Development, LLC, Spring House, PA, United States
Meijian Guan	Janssen Research & Development, LLC, Spring House, PA, United States
Shicheng Guo	Janssen Research & Development, LLC, Spring House, PA, United States
Beryl Cummings	Maze Therapeutics, San Francisco, CA, United States
Matt Brauer	Maze Therapeutics, San Francisco, CA, United States
Jason Miller	Merck, Kenilworth, NJ, United States
Fabiana Farias	Merck, Kenilworth, NJ, United States
Jorge Del-Aguila	Merck, Kenilworth, NJ, United States
Kirill Shkura	Merck, Kenilworth, NJ, United States
Victor Neduva	Merck, Kenilworth, NJ, United States
Huilei Xu	Novartis, Basel, Switzerland
Amy Cole	Novartis, Basel, Switzerland
Jonathan Chung	Novartis, Basel, Switzerland
Jaison Jacob	Novartis, Basel, Switzerland
Katrina de Lange	Novartis, Basel, Switzerland
Jonas Zierer	Novartis, Basel, Switzerland
Xing Chen	Pfizer, New York, NY, United States
Åsa Hedman	Pfizer, New York, NY, United States
Clarence Wang	Sanofi, Paris, France
Ethan Xu	Sanofi, Paris, France
Franck Auge	Sanofi, Paris, France
Clement Chatelain	Sanofi, Paris, France
Deepak Rajpal	Sanofi, Paris, France

Large Registry Based Analysis of Genetic Predisposition to Tuberculosis
Identifies Genetic Risk Factors at HLA
Supplements

Dongyu Liu	Sanofi, Paris, France
Katherine Call	Sanofi, Paris, France
Tai-He Xia	Sanofi, Paris, France
Mitja Kurki	Institute for Molecular Medicine Finland, HiLIFE, University of Helsinki, Finland / Broad Institute, Cambridge, MA, United States
Samuli Ripatti	Institute for Molecular Medicine Finland, HiLIFE, University of Helsinki, Finland
Mark Daly	Institute for Molecular Medicine Finland, HiLIFE, University of Helsinki, Finland
Juha Karjalainen	Institute for Molecular Medicine Finland, HiLIFE, University of Helsinki, Finland
Aki Havulinna	Institute for Molecular Medicine Finland, HiLIFE, University of Helsinki, Finland
Juha Mehtonen	Institute for Molecular Medicine Finland, HiLIFE, University of Helsinki, Finland
Priit Palta	Institute for Molecular Medicine Finland, HiLIFE, University of Helsinki, Finland
Shabbeer Hassan	Institute for Molecular Medicine Finland, HiLIFE, University of Helsinki, Finland
Pietro Della Briotta Parolo	Institute for Molecular Medicine Finland, HiLIFE, University of Helsinki, Finland
Wei Zhou	Broad Institute, Cambridge, MA, United States
Mutaamba Maasha	Broad Institute, Cambridge, MA, United States
Shabbeer Hassan	Institute for Molecular Medicine Finland, HiLIFE, University of Helsinki, Finland
Susanna Lemmelä	Institute for Molecular Medicine Finland, HiLIFE, University of Helsinki, Finland
Manuel Rivas	University of Stanford, Stanford, CA, United States
Aarno Palotie	Institute for Molecular Medicine Finland, HiLIFE, University of Helsinki, Finland
Arto Lehisto	Institute for Molecular Medicine Finland, HiLIFE, University of Helsinki, Finland
Andrea Ganna	Institute for Molecular Medicine Finland, HiLIFE, University of Helsinki, Finland

Large Registry Based Analysis of Genetic Predisposition to Tuberculosis
Identifies Genetic Risk Factors at HLA
Supplements

Vincent Llorens	Institute for Molecular Medicine Finland, HiLIFE, University of Helsinki, Finland
Hannele Laivuori	Institute for Molecular Medicine Finland, HiLIFE, University of Helsinki, Finland
Taru Tukiainen	Institute for Molecular Medicine Finland, HiLIFE, University of Helsinki, Finland
Mary Pat Reeve	Institute for Molecular Medicine Finland, HiLIFE, University of Helsinki, Finland
Henrike Heyne	Institute for Molecular Medicine Finland, HiLIFE, University of Helsinki, Finland
Nina Mars	Institute for Molecular Medicine Finland, HiLIFE, University of Helsinki, Finland
Kimmo Palin	University of Helsinki, Helsinki, Finland
Javier Garcia-Tabuenca	University of Tampere, Tampere, Finland
Harri Siirtola	University of Tampere, Tampere, Finland
Tuomo Kiiskinen	Institute for Molecular Medicine Finland, HiLIFE, University of Helsinki, Finland
Jiwoo Lee	Institute for Molecular Medicine Finland, HiLIFE, University of Helsinki, Finland / Broad Institute, Cambridge, MA, United States
Kristin Tsuo	Institute for Molecular Medicine Finland, HiLIFE, University of Helsinki, Finland / Broad Institute, Cambridge, MA, United States
Amanda Elliott	Institute for Molecular Medicine Finland, HiLIFE, University of Helsinki, Finland / Broad Institute, Cambridge, MA, United States
Kati Kristiansson	THL Biobank / The National Institute of Health and Welfare Helsinki, Finland
Mikko Arvas	Finnish Red Cross Blood Service / Finnish Hematology Registry and Clinical Biobank, Helsinki, Finland
Kati Hyvärinen	Finnish Red Cross Blood Service, Helsinki, Finland
Jarmo Ritari	Finnish Red Cross Blood Service, Helsinki, Finland
Miika Koskinen	Helsinki Biobank / Helsinki University and Hospital District of Helsinki and Uusimaa, Helsinki
Olli Carpén	Helsinki Biobank / Helsinki University and Hospital District of Helsinki and Uusimaa, Helsinki
Johannes Kettunen	Northern Finland Biobank Borealis / University of Oulu / Northern Ostrobothnia Hospital District, Oulu, Finland

Large Registry Based Analysis of Genetic Predisposition to Tuberculosis
Identifies Genetic Risk Factors at HLA
Supplements

Katri Pylkäs	University of Oulu, Oulu, Finland
Eeva Sliz	University of Oulu, Oulu, Finland
Minna Karjalainen	University of Oulu, Oulu, Finland
Tuomo Mantere	Northern Finland Biobank Borealis / University of Oulu / Northern Ostrobothnia Hospital District, Oulu, Finland
Eeva Kangasniemi	Finnish Clinical Biobank Tampere / University of Tampere / Pirkanmaa Hospital District, Tampere, Finland
Sami Heikkinen	University of Eastern Finland, Kuopio, Finland
Arto Mannermaa	Biobank of Eastern Finland / University of Eastern Finland / Northern Savo Hospital District, Kuopio, Finland
Eija Laakkonen	University of Jyväskylä, Jyväskylä, Finland
Dhanaprakash Jambulingam	University of Turku, Turku, Finland
Venkat Subramaniam Rathinakannan	University of Turku, Turku, Finland
Nina Pitkänen	Auria Biobank / University of Turku / Hospital District of Southwest Finland, Turku, Finland

Biobank directors

Lila Kallio	Auria Biobank / University of Turku / Hospital District of Southwest Finland, Turku, Finland
Sirpa Soini	THL Biobank / The National Institute of Health and Welfare Helsinki, Finland
Jukka Partanen	Finnish Red Cross Blood Service / Finnish Hematology Registry and Clinical Biobank, Helsinki, Finland
Eero Punkka	Helsinki Biobank / Helsinki University and Hospital District of Helsinki and Uusimaa, Helsinki
Raisa Serpi	Northern Finland Biobank Borealis / University of Oulu / Northern Ostrobothnia Hospital District, Oulu, Finland
Sanna Siltanen	Finnish Clinical Biobank Tampere / University of Tampere / Pirkanmaa Hospital District, Tampere, Finland
Veli-Matti Kosma	Biobank of Eastern Finland / University of Eastern Finland / Northern Savo Hospital District, Kuopio, Finland

Teijo Kuopio	Central Finland Biobank / University of Jyväskylä / Central Finland Health Care District, Jyväskylä, Finland

FinnGen Teams

Administration

Anu Jalanko	Institute for Molecular Medicine Finland, HiLIFE, University of Helsinki, Finland
Huei-Yi Shen	Institute for Molecular Medicine Finland, HiLIFE, University of Helsinki, Finland
Risto Kajanne	Institute for Molecular Medicine Finland, HiLIFE, University of Helsinki, Finland
Mervi Aavikko	Institute for Molecular Medicine Finland, HiLIFE, University of Helsinki, Finland

Analysis

Mitja Kurki	Institute for Molecular Medicine Finland, HiLIFE, University of Helsinki, Finland / Broad Institute, Cambridge, MA, United States
Juha Karjalainen	Institute for Molecular Medicine Finland, HiLIFE, University of Helsinki, Finland
Pietro Della Briotta Parolo	Institute for Molecular Medicine Finland, HiLIFE, University of Helsinki, Finland
Arto Lehisto	Institute for Molecular Medicine Finland, HiLIFE, University of Helsinki, Finland
Juha Mehtonen	Institute for Molecular Medicine Finland, HiLIFE, University of Helsinki, Finland
Wei Zhou	Broad Institute, Cambridge, MA, United States
Masahiro Kanai	Broad Institute, Cambridge, MA, United States
Mutaamba Maasha	Broad Institute, Cambridge, MA, United States

Clinical Endpoint Development

Hannele Laivuori	Institute for Molecular Medicine Finland, HiLIFE, University of Helsinki, Finland

Large Registry Based Analysis of Genetic Predisposition to Tuberculosis
Identifies Genetic Risk Factors at HLA
Supplements

Aki Havulinna Institute for Molecular Medicine Finland, HiLIFE, University of Helsinki, Finland

Susanna Lemmelä Institute for Molecular Medicine Finland, HiLIFE, University of Helsinki, Finland

Tuomo Kiiskinen Institute for Molecular Medicine Finland, HiLIFE, University of Helsinki, Finland

L. Elisa Lahtela Institute for Molecular Medicine Finland, HiLIFE, University of Helsinki, Finland

Communication

Mari Kaunisto Institute for Molecular Medicine Finland, HiLIFE, University of Helsinki, Finland

E-Science

Elina Kilpeläinen Institute for Molecular Medicine Finland, HiLIFE, University of Helsinki, Finland

Timo P. Sipilä Institute for Molecular Medicine Finland, HiLIFE, University of Helsinki, Finland

Oluwaseun Alexander Dada Institute for Molecular Medicine Finland, HiLIFE, University of Helsinki, Finland

Awaisa Ghazal Institute for Molecular Medicine Finland, HiLIFE, University of Helsinki, Finland

Anastasia Shcherban Institute for Molecular Medicine Finland, HiLIFE, University of Helsinki, Finland

Rigbe Weldatsadik Institute for Molecular Medicine Finland, HiLIFE, University of Helsinki, Finland

Genotyping

Kati Donner Institute for Molecular Medicine Finland, HiLIFE, University of Helsinki, Finland

Timo P. Sipilä Institute for Molecular Medicine Finland, HiLIFE, University of Helsinki, Finland

Sample Collection Coordination

Large Registry Based Analysis of Genetic Predisposition to Tuberculosis
Identifies Genetic Risk Factors at HLA
Supplements

Anu Loukola	Helsinki Biobank / Helsinki University and Hospital District of Helsinki and Uusimaa, Helsinki

Sample Logistics

Päivi Laiho	THL Biobank / The National Institute of Health and Welfare Helsinki, Finland
Tuuli Sistonen	THL Biobank / The National Institute of Health and Welfare Helsinki, Finland
Essi Kaiharju	THL Biobank / The National Institute of Health and Welfare Helsinki, Finland
Markku Laukkanen	THL Biobank / The National Institute of Health and Welfare Helsinki, Finland
Elina Järvensivu	THL Biobank / The National Institute of Health and Welfare Helsinki, Finland
Sini Lähteenmäki	THL Biobank / The National Institute of Health and Welfare Helsinki, Finland
Lotta Männikkö	THL Biobank / The National Institute of Health and Welfare Helsinki, Finland
Regis Wong	THL Biobank / The National Institute of Health and Welfare Helsinki, Finland

Registry Data Operations

Hannele Mattsson	THL Biobank / The National Institute of Health and Welfare Helsinki, Finland
Kati Kristiansson	THL Biobank / The National Institute of Health and Welfare Helsinki, Finland
Susanna Lemmelä	Institute for Molecular Medicine Finland, HiLIFE, University of Helsinki, Finland
Sami Koskelainen	THL Biobank / The National Institute of Health and Welfare Helsinki, Finland
Tero Hiekkalinna	THL Biobank / The National Institute of Health and Welfare Helsinki, Finland
Teemu Paajanen	THL Biobank / The National Institute of Health and Welfare Helsinki, Finland

Large Registry Based Analysis of Genetic Predisposition to Tuberculosis
Identifies Genetic Risk Factors at HLA
Supplements

Sequencing Informatics

Priit Palta	Institute for Molecular Medicine Finland, HiLIFE, University of Helsinki, Finland
Kalle Pärn	Institute for Molecular Medicine Finland, HiLIFE, University of Helsinki, Finland
Mart Kals	Institute for Molecular Medicine Finland, HiLIFE, University of Helsinki, Finland
Shuang Luo	Institute for Molecular Medicine Finland, HiLIFE, University of Helsinki, Finland
Vishal Sinha	Institute for Molecular Medicine Finland, HiLIFE, University of Helsinki, Finland

Trajectory

Tarja Laitinen	Pirkanmaa Hospital District, Tampere, Finland
Mary Pat Reeve	Institute for Molecular Medicine Finland, HiLIFE, University of Helsinki, Finland
Marianna Niemi	University of Tampere, Tampere, Finland
Harri Siirtola	University of Tampere, Tampere, Finland
Javier Gracia-Tabuenca	University of Tampere, Tampere, Finland
Mika Helminen	University of Tampere, Tampere, Finland
Tiina Luukkaala	University of Tampere, Tampere, Finland
Iida Vähätalo	University of Tampere, Tampere, Finland

Data protection officer

Jyrki Pitkänen	Institute for Molecular Medicine Finland, HiLIFE, University of Helsinki, Finland

FINBB - Finnish biobank cooperative

Marco Hautalahti

Johanna Mäkelä

Mirkka Koivusalo

Large Registry Based Analysis of Genetic Predisposition to Tuberculosis
Identifies Genetic Risk Factors at HLA
Supplements

Sarah Smith

Tom Southerington
